# Concerted Metabolic Shifts Give New Insights Into the Syntrophic Mechanism Between Propionate-Fermenting *Pelotomaculum thermopropionicum* and Hydrogenotrophic *Methanocella conradii*

**DOI:** 10.3389/fmicb.2018.01551

**Published:** 2018-07-09

**Authors:** Pengfei Liu, Yahai Lu

**Affiliations:** College of Urban and Environmental Sciences, Peking University, Beijing, China

**Keywords:** *P. thermopropionicum*, *M. conradii*, syntrophy, propionate, formate, interspecies electron transfer, RNA-Seq

## Abstract

Microbial syntrophy is a thermodynamically-based cooperation between microbial partners that share the small amounts of free energy for anaerobic growth. To gain insights into the mechanism by which syntrophic microorganisms coordinate their metabolism, we constructed cocultures of propionate-oxidizing *Pelotomaculum thermopropionicum* and hydrogenotrophic *Methanocella conradii* and compared them to monocultures. Transcriptome analysis was performed on these cultures using strand-specific mRNA sequencing (RNA-Seq). The results showed that in coculture both *P. thermopropionicum* and *M. conradii* significantly upregulated the expression of genes involved in catabolism but downregulated those for anabolic biosynthesis. Specifically, genes coding for the methylmalonyl-CoA pathway in *P. thermopropionicum* and key genes for methanogenesis in *M. conradii* were substantially upregulated in coculture compared to monoculture. The putative flavin-based electron bifurcation/confurcation systems in both organisms were also upregulated in coculture. Formate dehydrogenase encoding genes in both organisms were markedly upregulated, indicating that formate was produced and utilized by *P. thermopropionicum* and *M. conradii*, respectively. The inhibition of syntrophic activity by formate and 2-bromoethanesulphonate (2-BES) but not H_2_/CO_2_ also suggested that formate production was used by *P. thermopropionicum* for the recycling of intracellular redox mediators. Finally, flagellum-induced signal transduction and amino acids exchange was upregulated for syntrophic interactions. Together, our study suggests that syntrophic organisms employ multiple strategies including global metabolic shift, utilization of electron bifurcation/confurcation and employing formate as an alternate electron carrier to optimize their metabolisms for syntrophic growth.

## Introduction

In anoxic habitats, microbial consortia, of which each member has a specific metabolic function, perform the degradation of complex organic matter to carbon dioxide (CO_2_) and methane (CH_4_) (Schink, 1997; Conrad, 1999). Syntrophic oxidation of key intermediates (e.g., propionate and butyrate) requires tightly coupled, mutualistic interactions between fermentative bacteria and methanogenic archaea (Schink and Stams, 2006; McInerney et al., 2008, 2009). How syntrophic consortia conserve energy from reactions that operate close to the thermodynamic equilibrium and which molecular mechanisms are involved in the establishment and maintenance of syntrophic interactions still are open questions (Sieber et al., 2012).

Propionate is next to acetate as an important intermediate during methanogenic degradation of organic matter in anoxic habitats (Rothfuss and Conrad, 1993; Glissmann and Conrad, 2000; McInerney et al., 2009). Two types of syntrophic bacteria are found to oxidize propionate in association with methanogens. The first type utilizes the methylmalonyl-CoA (MMC) pathway in which three pairs of electrons from propionate oxidation are released in the form of hydrogen (H_2_) and/or formate. Organisms of this type include *Syntrophobacter* spp. and *Pelotomaculum* spp. (McInerney et al., 2008; Müller et al., 2010). The second type oxidizes propionate through a dismutating pathway in which two propionate molecules are first jointed to form a C_6_ compound which is then dismutated to acetate and butyrate before being degraded via beta oxidation. The main organisms of this type include *Smithella* spp. (de Bok et al., 2001).

*Pelotomaculum* spp. are widespread in bioreactors (Imachi et al., 2002; Nobu et al., 2015) and in natural environments like rice field soils (Lueders et al., 2004; Gan et al., 2012) where they cooperate with methanogens to oxidize propionate and other organic compounds. *Pelotomaculum thermopropionicum* has been used as a model organism for studies of the physiology and ecology of propionate-oxidizing syntrophs (Ishii et al., 2005, 2006; Kato et al., 2009). The genome of *P. thermopropionicum* reveals that several peripheral pathways are associated with the central MMC pathway, indicating that these organisms can have a broad substrate specificity (Imachi et al., 2002; Kosaka et al., 2006, 2008). Genes coding for most of the MMC pathway form a large gene cluster (the *mmc* cluster), which may facilitate its coordinate expression and hence save transcription costs (Kosaka et al., 2006, 2008). The genome encodes four hydrogenases and two formate dehydrogenases, some of which are considered to be capable of mediating electron confurcation (Kosaka et al., 2008; Kato et al., 2009; Sieber et al., 2010). In addition, the genome encodes a large number of signal-transduction mechanisms (Kosaka et al., 2008). Transcriptomic analysis revealed that *P. thermopropionicum* has complex regulatory mechanisms for gene expression and for metabolism in response to substrate addition and coculture conditions (Kato et al., 2009). There is also evidence that *P. thermopropionicum* may be capable of flagellum-mediated communication with the methanogen partners (Shimoyama et al., 2009).

Different methanogens have been used for studies on syntrophic metabolism (Kato et al., 2009; Enoki et al., 2011; Worm et al., 2011b; Walker et al., 2012; Sieber et al., 2015). *Methanocella* spp. (order *Methanocellales*), of which so far three strains were isolated, were all obtained from paddy field soil (Sakai et al., 2008, 2010; Lü and Lu, 2012). However, environmental surveys indicate that *Methanocellales* are widespread in nature including not only paddy field soil but also peat bogs, river and lake sediment, and even upland and desert soils (Conrad et al., 2006; Angel et al., 2011, 2012; Aschenbach et al., 2013). *Methanocella* spp. are hydrogenotrophic and well-adapted to low H_2_ concentrations (Lu et al., 2005; Sakai et al., 2009). Consequently, they are often detected in association with secondary fermenters for syntrophic oxidation of fatty acids (e.g., propionate) occurring as intermediates of the anaerobic degradation of organic matter in paddy field soil (Lueders et al., 2004; Liu and Conrad, 2010; Liu P. et al., 2011; Rui et al., 2011; Gan et al., 2012). The genome analysis of *Methanocella* spp. has reconstructed their core metabolism and predicted certain novel features, including a set of genes potentially involved in utilizing low concentrations of H_2_ (Lyu and Lu, 2015). A novel hydrogenase, tentatively named as disulfide-reducing hydrogenase (Drh), was discovered that is phylogenetically more closely related to Coo hydrogenase (carbon monoxide-induced hydrogenase) of bacterial sulfate reducers than to any canonical hydrogenase of archaeal methanogens (Lyu and Lu, 2015). We have previously constructed syntrophic cocultures using *Methanocella conradii* in combination with either *Syntrophomonas wolfei* or *P. thermopropionicum* (Liu et al., 2014), which revealed that *M.conradii* in coculture tended to upregulate the expression of genes coding for methanogenesis pathway but downregulate the genes coding for anabolic biosynthesis. However, only a few functional genes were examined and moreover the response of the bacterial partners was not evaluated. Therefore, a holistic understanding on how syntrophic organisms coordinate their metabolism under energy-limiting conditions is still lacking.

Both hydrogen and formate transfer are important interspecies electron transfer mechanisms (de Bok et al., 2004; Sieber et al., 2012; Schink et al., 2017; Sedano-Núñez et al., 2018). It has been reported that formate is temporarily produced by *P. thermopropionicum* when it grows on ethanol under syntrophic association with a hydrogenotrophic methanogen (Kosaka et al., 2008). However, it is still unclear whether *P. thermopropionicum* also produces formate when growing on propionate. Although this possibility has been suggested to be unlikely (Imachi et al., 2002), a significant upregulation of formate dehydrogenases was observed in *P. thermopropionicum* when grown in propionate degrading coculture (Kato et al., 2009). *M. conradii* cannot use formate as sole substrate for growth and methane production (Lü and Lu, 2012). However, we previously showed that *M. conradii* can use formate for methane production if H_2_ is supplied in addition. Furthermore, formate dehydrogenase was upregulated in syntrophic cocultures with propionate- or butyrate- degrading syntrophs, indicating that *M. conradii* is able to perform formate-dependent methanogenesis (Liu et al., 2014). However, since such upregulation may be merely a response to H_2_ limitation (Nölling and Reeve, 1997; Wood et al., 2003; Hendrickson et al., 2007), the importance of formate as electron shuttle between *P. thermopropionicum* and *M. conradii* is still not clear.

In the present study, high throughput sequencing of transcriptomes (RNA-Seq) was used to study the global gene expression in a coculture of *P. thermopropionicum* and *M. conradii*. RNA-Seq is believed to offer a greater dynamic range with less background noise and reveal sequence identity directly (Giannoukos et al., 2012; McGettigan, 2013; Hrdlickova et al., 2017). The strand-specific RNA-Seq in particular resolves the correct expression levels of coding or non-coding over-lapping transcripts and provides accurate quantification of gene expression (Levin et al., 2010). We hypothesized that interactions during syntrophic growth will trigger global transcriptional responses in both *P. thermopropionicum* and *M. conradii*. A further aspect was the role of formate in interspecies electron transfer.

## Materials and methods

### Cultivation and cell collection

*P. thermopropionicum* strain SI^T^ (DSM 13744) monoculture (abbreviated as PM) was grown in basal medium with 20 mM pyruvate as substrate under N_2_/CO_2_ (80/20, v/v) at 170 kPa (Imachi et al., 2002; Liu et al., 2014). *M. conradii* strain HZ254^T^ (DSM 24964) was grown in monoculture (abbreviated as MM) under H_2_/CO_2_ (80/20, v/v) at 170 kPa or in coculture (abbreviated as PMC) with *P. thermopropionicum* by using 20 mM propionate as substrate under N_2_/CO_2_ (80/20, v/v) at 170 kPa, as previously described (Lü and Lu, 2012; Liu et al., 2014). All the components of the basal medium were the same for all cultures except for the substrates. To obtain adequate amounts of RNA for sequencing, the culture volume was 100 ml in 550-ml bottle for PM, 500 ml in 2.3-L bottle for MM, and 500 ml in 1.1-L bottle for PMC. Cultivation was carried out at 55°C in the dark without agitation. The growth of PM was monitored by measuring optical density at 578 nm (OD578) and those of MM and PMC were monitored by analysis of CH_4_ production in the headspace as previously described (Liu et al., 2014). All cultures were harvested for transcriptomic analysis at the mid- to late exponential growth phases (Figure S1). Cell suspensions were centrifuged at 23,000 g at 4°C for 20 min (Avanti J-26XP, Beckman Coulter, USA), supernatants were discarded and 1.8 ml RNAlater (Ambion, USA) was added to the pellets immediately, which were then incubated at 4°C overnight. Cells were recollected by centrifuging at 20,000 g at 4°C for 60 min. Supernatants were discarded carefully and the pellets were stored immediately at −80°C until RNA extraction.

In order to test the effects of 2-BES, H_2_/CO_2_ and formate on PMC, 100 ml cultures in 250-ml bottle were used. 2-BES served as a specific methanogenic inhibitor (Liu H. et al., 2011). Other culture conditions were the same as described above. Consumption of propionate, H_2_, and formate, and the production of CH_4_ and acetate were monitored by GC or HPLC as previously described (Liu et al., 2014). Five mM 2-BES, 20 mM formate and/or H_2_/CO_2_ (80/20, v/v, 170 kPa) were added after the initiation of propionate degradation. The headspace of the bottles treated with 20 mM formate were re-flushed with N_2_/CO_2_ (80/20, v/v) at 170 kPa.

### Total RNA extraction and mRNA enrichment

Total RNA was extracted using AllPrep DNA/RNA Mini Kit (Qiagen, Germany) according to the manufacturer's instruction. Lysis buffer RLT plus was supplemented with 2% (v/v) 2 M Dithiothreitol (DTT) (Sangon Biotech, China). Lysis buffer RLT plus (600 μl) was added to each cell pellet. Cell pellets of PM and PMC were homogenized for 120 s (5.0 m/s, 3 × 40 s with 2 min intervals on ice) in a FastPrep-24 (MP Biomedicals, USA) with 0.5 g RNase free beating beads (0.1 mm, Sigma, USA). Cell pellets of MM were homogenized at 5.0 m/s for 20 s without beating beads. Residue DNA digestion, verification of absence of DNA, and RNA purification were carried out as previously described (Liu et al., 2014). RNA was finally dissolved in 75 μl RNase free H_2_O. RNA integrity values (RIN) were determined by an Agilent 2100 Bioanalyzer (Agilent Technologies, USA) according to the manufacturer's instruction (Figure S2). Aliquots of total RNA were kept for quantitative real-time PCR (qRT-PCR), and the remainder (≥10 μg) was sent to Beijing Genomics Institute (BGI) (http://www.genomics.cn, China) on dry ice for mRNA enrichment by using the Ribo-Zero™ Magnetic Kit for Bacteria (Epicenter, USA).

### Strand-specific library construction, illumina sequencing, and data analysis

RNA fragmentation, strand-specific cDNA synthesis (Parkhomchuk et al., 2009), library preparation, sequencing (2 × 90 paired-end reads on an Illumina HiSeq 2000 sequencer) and base calling were performed by BGI according to their in-house procedures (Liu L. et al., 2011). Four biological replicates were sequenced for PM, MM and PMC, respectively, yielding ≥ 25 million clean paired-end reads per replicate (Table S1). Reads were mapped to reference genomes using the function BWA-MEM of bwa program version 0.7.5a-r405 (Li and Durbin, 2009) with parameters: -t 4. Uniquely mapped reads were filtered by using samtools-0.1.19 (Li et al., 2009), with parameters: -f 0 × 2, -q 30, -F 0 × 100, NM ≤ 5. On average, 0.10, 6.74, and 4.98% of the total reads were mapped to rRNAs for PM, MM and PMC, respectively. These rRNA reads were excluded for further analysis (Table S1). To exclude reads that may map ambiguously (that is, to either genome), we did a cross reference mapping by using the parameter described above (Figure S3). Mapping of reads from PM and MM to the opposite genome showed only 0.62–1.42% and ~0.1% of the total reads from PM and MM, respectively (Table S1). These reads were filtered out (masked from downstream analysis). Unmapped reads from PM and MM were mapped to the genome of *P. thermopropionicum* (dataset PMp) and *M. conradii* (dataset MMm), respectively. For PMC, reads were mapped to the genome of either *M. conradii* or *P. thermopropionicum*. Reads unmapped to *M. conradii* were mapped to the genome *P. thermopropionicum* (dataset PMCp) and reads unmapped to the genome of *P. thermopropionicum* were mapped to the genome of *M. conradii* (dataset PMCm), respectively. Library strand specificity was checked by using RSeQC (Wang et al., 2012) (Table S2). Unique mapped reads were analyzed and assigned to individual CDS (coding DNA sequence) by HTSeq (Anders et al., 2015) according to the genome annotation provided by GenBank (*P. thermopropionicum*: NC_009454 and *M. conradii*: NC_017034). Following parameters set for HTSeq were: -m union, –nonunique = none, -t gene, -i locus_tag, -s reverse (strand specificity libraries, according to the results of RSeQC).

DESeq2 package (version 1.6.2) in R (R Core Team, 2016) was then used to assess differential expression across the transcriptome (Love et al., 2014). The function DESeq was called with design formula “~condition,” in which monoculture conditions of either *M. conradii* or *P. thermopropionicum* were used as control groups and the coculture were used as treatment groups. DESeq2 performs a hypothesis test on each gene to see whether evidence is sufficient to decide against the *null hypothesis* that there is no effects of the coculture on the gene expression and that the observed difference between coculture and monoculture was merely caused by experimental variability. Only absolute log_2_-fold change values (L2fc) ≥ 1.0 with false discovery rate (FDR) < 0.05 in transcription were considered significant. Principal components analysis (PCA) was performed using the function “prcomp” in R using the variance stabilizing transformed data from DESeq2. “Fragments Per Kilobase of CDS (or exon) per Million reads” (FPKM) was calculated according to the below formula (Trapnell et al., 2010):

FPKM = Raw counts × 10^9^/(length of that CDS × total number of mapped reads).

The pearson correlation coefficients based on log_2_ FPKM value of each CDS were calculated, which showed that biological replicates within each group were reproducible (Table S3). Furthermore, we calculated the log_2_ value of mean FPKM (L2mFPKM) for each dataset, with the median of 6.9, 6.8, 6.7, and 6.3 for PMp, PMCp, MMm, and PMCm, respectively. The median was used as an indication of expression activity (Shrestha et al., 2013).

### qRT-PCR verification of transcriptomic data

To verify the differential expression level detected by sequencing, qRT-PCR was performed for 6 differential expressed genes of *P. thermopropionicum* and *M. conradii*, respectively. Primers targeting the selected genes were designed by using Primer Premier 6 (Premier, Canada) and synthesized by Life Technologies (Shanghai, China) (Table S4). cDNA synthesis was carried out as previously described (Liu et al., 2014). qRT-PCR and data analysis was performed with the 7500 real-time PCR system (Applied Biosystems, USA) (Liu et al., 2014). The expression levels of 16S rRNA gene and DNA-directed RNA polymerase beta subunit encoding gene (*rpoC*) of *P. thermopropionicum* and 16S rRNA gene and DNA-directed RNA polymerase A subunit encoding gene (*rpoA1*) of *M. conradii* were chosen as the references, respectively.

### Retrieval of microbial signal transduction system (STS) related genes

Genes related to signal transduction in *P. thermopropionicum* and *M. conradii* were retrieved from the microbial signal transduction database (MiST 2.2, http://mistdb.com/) (Ulrich and Zhulin, 2010).

### Nucleotide sequence accession number

The sequence reads determined in this study have been submitted to the GEO databases under accession number GSE103596.

## Results

### Overview of RNA-seq transcriptomes

In monoculture, *P. thermopropionicum* on pyruvate and *M. conradii* on H_2_/CO_2_ grew with generation times of 6–7 and 7–8 h, respectively. In syntrophic coculture on propionate, generation times were in a range of 36–40 h (Figure S1). These generation times were consistent with those obtained in previous studies (Imachi et al., 2002; Lü and Lu, 2012; Liu et al., 2014).

Of all the sequenced RNA-Seq reads, 75.6 and 78.0% were mapped to coding DNA sequences (CDSs) of the corresponding genomes of all reads from PM and MM, respectively. Of the reads from PMC, 9.3 and 68.4% were mapped to *P. thermopropionicum* and *M. conradii* CDSs, respectively (Table S1). The remaining reads were mapped to genome regions without annotation or with reverse complemented directions (antisense) to CDSs (data not shown).

**Figure 1:**
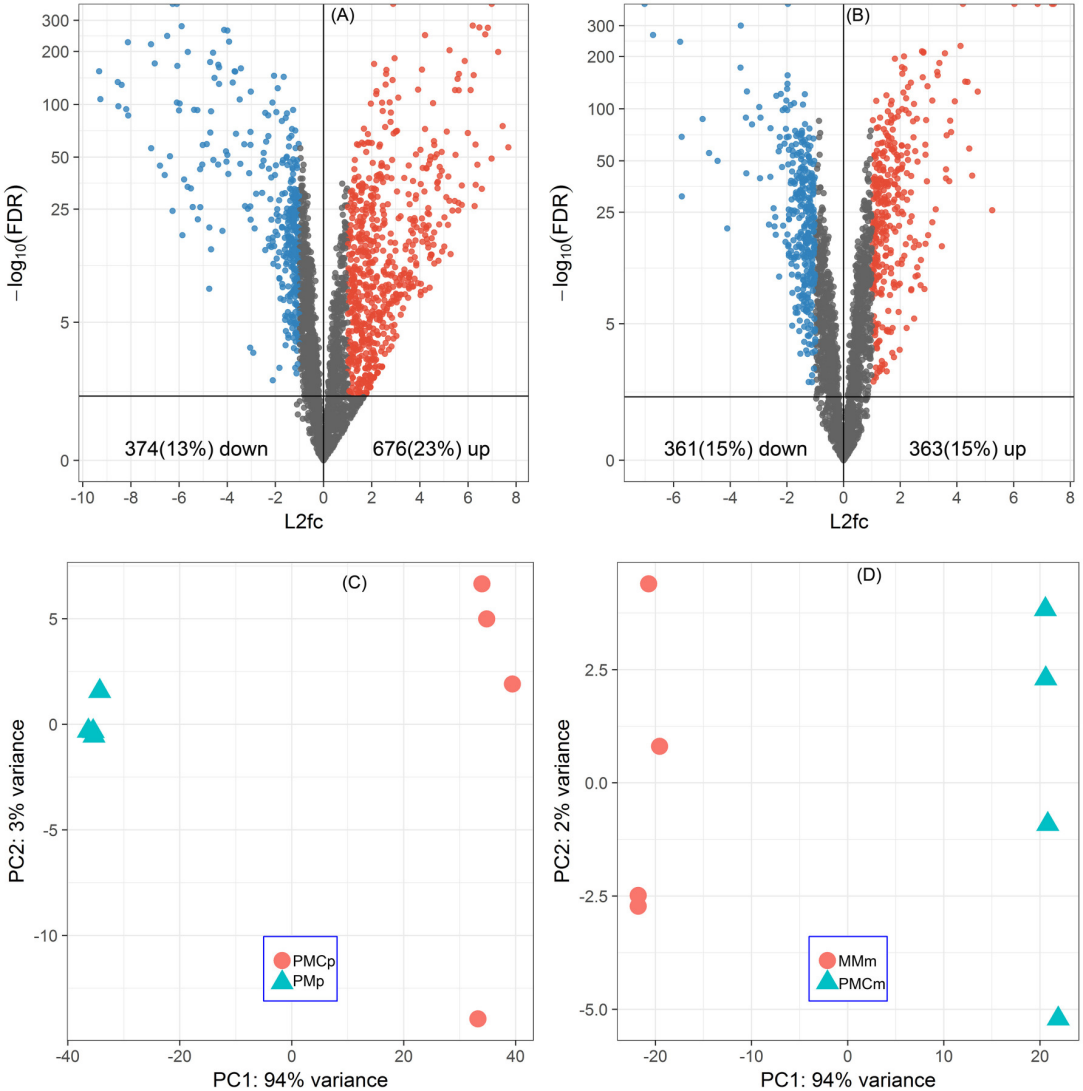
Global responses of *P. thermopropionicum* and *M. conradii* to syntrophic coculture conditions. Volcano plots of differential transcription levels of *P. thermopropionicum* **(A)** and *M. conradii* **(B)** in coculture. Genes with absolute log_2_-fold change value (L2fc) ≥ 1 and false discovery rate (FDR) < 0.05 are considered as significantly regulated (red for up and blue for down). Results of principal component analysis (PCA) based on all DESeq2 variance transformed transcripts abundance data of *P. thermopropionicum* **(C)** and *M. conradii* **(D)**. PMp, dataset of *P. thermopropionicum* monoculture; PMCp, dataset of syntrophic coculture mapped to *P. thermopropionicum* genome; MMm, dataset of *M. conradii* monoculture; and PMCm, dataset of syntrophic coculture mapped to *M. conradii* genome.

Of all the 2920 CDSs of *P. thermopropionicum* and 2455 CDSs of *M. conradii*, 676 (23%) and 363 (15%) were significantly upregulated and 374 (13%) and 361 (15%) were significantly downregulated in the coculture compared to the respective monocultures (Figures 1A,B and Supplementary Datasets Pt1, Mc1). Principal component analysis (PCA) analysis showed that the gene expression profiles were distinct between monoculture and coculture conditions (Figures 1C,D). Differential expression of selected genes determined by qRT-PCR correlated well with that by RNA-Seq (Pearson *r* > 0.99) and had a slope that approached one (Figure S4).

In order to evaluate global transcriptional responses of *P. thermopropionicum* and *M. conradii* to syntrophic growth, the numbers of differentially expressed genes were summarized as functional categories according to the clusters of orthologous groups of proteins (COGs) database (Tatusov et al., 2003) (Table 1). For both *P. thermopropionicum* and *M. conradii*, the upregulated genes in coculture were mainly distributed in “Energy production and conversion (C),” “Cell wall/membrane/envelope biogenesis (M),” “Carbohydrate transport and metabolism (G),” and “Signal transduction mechanisms (T).” The genes downregulated in coculture were mainly categorized into “Amino acid transport and metabolism (E)” and “Translation, ribosomal structure, and biogenesis (J)” (Table 1). In addition, a relatively large number of *P. thermopropionicum* genes belonging to “Replication, recombination, and repair (L),” “Lipid transport and metabolism (I),” and “Cell motility (N)” were upregulated in coculture.

**Table 1:** Distribution of differential expressed genes in clusters of orthologous groups (COGs) categories.

**COG categories**	***P. thermopropionicum***			***M. conradii***		
	**Total**	**Up^a^**	**Down^b^**	**Total**	**Up^a^**	**Down^b^**
**Energy production and conversion (C)^c^**	181	53^c^	33	173	29	15
**Translation, ribosomal structure and biogenesis (J)**	138	3	37^c^	163	5	86
**Amino acid transport and metabolism (E)**	183	30	47	147	14	29
Coenzyme transport and metabolism (H)	122	15	19	133	19	20
Inorganic ion transport and metabolism (P)	86	21	24	113	11	10
Transcription (K)	139	29	20	100	19	25
**Replication, recombination and repair (L)**	181	38	17	97	9	7
**Cell wall/membrane/envelope biogenesis (M)**	145	44	16	90	17	2
**Carbohydrate transport and metabolism (G)**	82	18	9	83	17	9
Posttranslational modification, protein turnover, chaperones (O)	78	17	11	83	8	12
**Signal transduction mechanisms (T)**	135	32	9	66	22	7
Nucleotide transport and metabolism (F)	66	6	10	54	9	7
Intracellular trafficking, secretion, and vesicular transport (U)	63	11	6	42	9	9
Cell cycle control, cell division, chromosome partitioning (D)	35	2	2	31	6	1
Defense mechanisms (V)	38	3	9	31	5	2
**Lipid transport and metabolism (I)**	71	22	5	22	3	9
**Cell motility (N)**	65	13	2	18	5	8
Cytoskeleton (Z)	n.a.	n.a.	n.a.	18	4	0
Secondary metabolites biosynthesis, transport and catabolism (Q)	26	6	3	5	0	2
Chromatin structure and dynamics (B)	3	1	0	3	0	2
RNA processing and modification (A)	n.a.	n.a.	n.a.	2	2	0
General function prediction only (R)	250	46	29	308	47	30
Function unknown (S)	191	38	29	249	35	34

*n.a., not applicable*.

a *log_2_-fold change value (L2fc) ≥ 1 and false discovery rate (FDR) < 0.05*.

b *L2fc ≤ −1 and FDR < 0.05*.

c *COG categories mentioned in the main text are shown in bold and red and blue colors depict that corresponding categories are featured by certain number of up or down regulated genes*.

### Metabolic shifts in *P. thermopropionicum*

**Figure 2:**
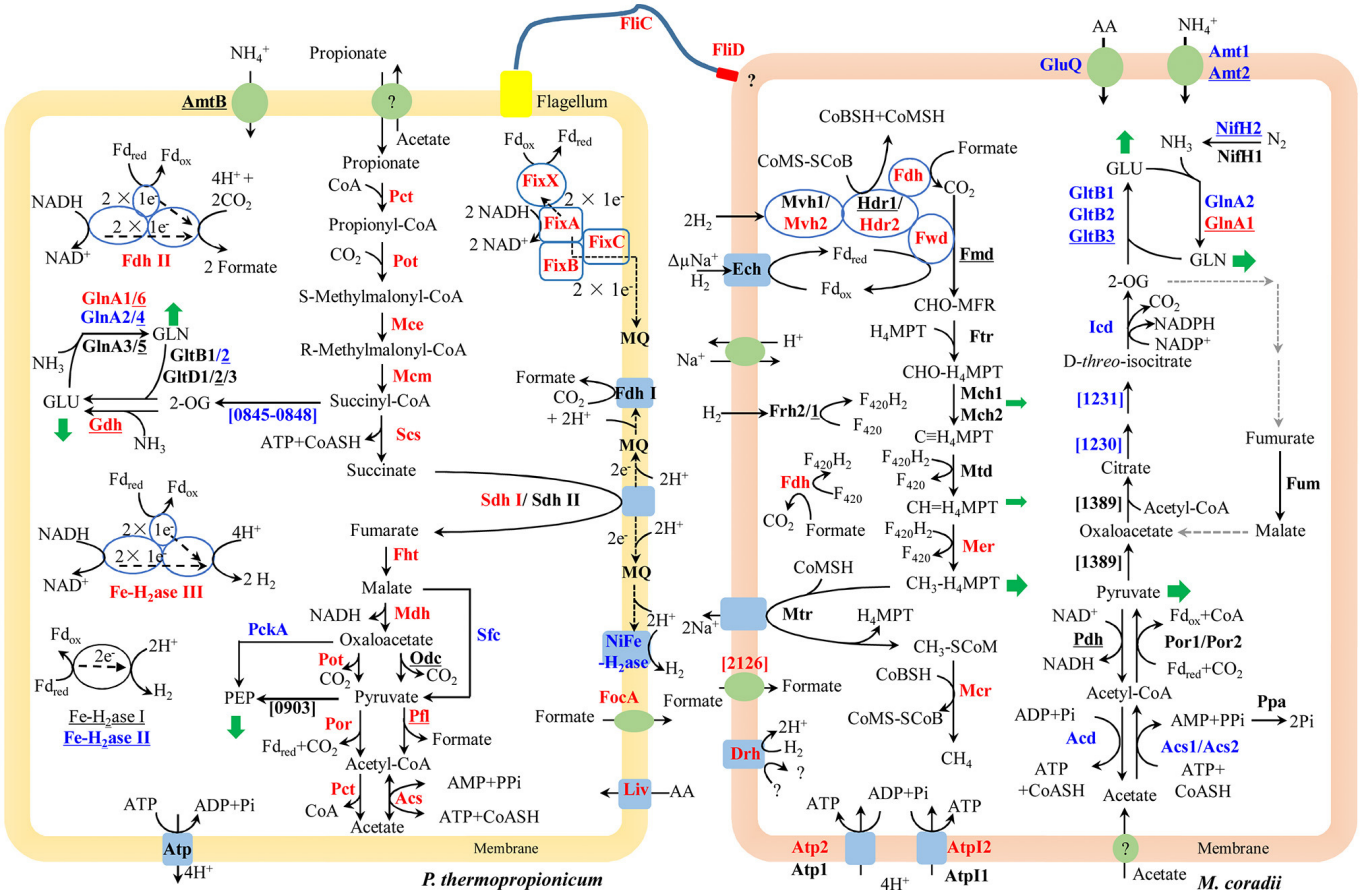
Conceptual metabolism scheme of *P. thermopropionicum* and *M. conradii* syntrophic coculture, highlighting the central energy conservation and early steps of biosynthesis pathways for both organisms. Enzymes catalyzing each step are shown in bold. Prefix omitted locus tags are shown for steps without a specific enzyme abbreviation. Significant changes (See Supplementary Dataset for details) in transcripts abundance during syntrophic growth are shown by red (up) and blue (down) coloration. Black coloration indicates statistically insignificant change. Monoculture of *P. thermopropionicum* and *M. conradii* served as control growth conditions. Enzymes encoded by genes with transcripts abundance lower than the median (as specified in the Materials and Methods section) of both monoculture and coculture conditions are underscored. Mvh2 and Hdr2 of *M. conradii* are also shown in red since coding genes for them and Fwd are in the same gene cluster (See Figure S9). Green arrows indicate intermediates for biosynthesis. Black and gray dash arrows indicate electron transfer and missing steps in the genome, respectively. Putative bifurcation and confurcation mediating complexes are shown in blue cycles. Abbreviation of enzyme names of *P. thermopropionicum* shown in the scheme: Pot, propionyl-CoA: oxaloacetate transcarboxylase; Mce, methylmalonyl-CoA epimerase; Mcm, methylmalonyl-CoA mutase; Scs, succinyl-CoA synthase; Fht, fumarase; Mdh, malate dehydrogenase; Por, pyruvate: ferredoxin oxidoreductase. Coding genes for above enzymes are assembled in the *mmc* gene cluster (PTH_1356-1369). Pct, acyl CoA: acetate/3-ketoacid CoA transferase; Sdh, succinate dehydrogenase (I & II); Acs, acetyl-CoA synthetases/AMP-(fatty) acid ligases; Odc, oxaloacetate decarboxylase; Pfl, pyruvate formate lyase; Fe-H_2_ase, Fe-hydrogenase (I, II & III,); NiFe-H_2_ase, NiFe-hydrogenase; Fdh, formate dehydrogenase (I & II); Fix complex, containing two subunits of the electron transfer flavoprotein (FixA and FixB), one ferredoxin-like protein (FixX), and one membrane-bound ETF-menaquinone oxidoreductase (FixC); AmtB, ammonia permease; GlnA, glutamine synthetase; Liv, ABC-type branched-chain amino acid transport systems. FliC, flagellin; FliD, flagellar capping protein; FocA, formate/nitrite family of transporters; Atp, F_0_F_1_-type ATP synthase; PckA, phosphoenolpyruvate carboxykinase; PTH_0903, phosphoenolpyruvate synthase/pyruvate phosphate dikinase; PTH_0845-0848, pyruvate: ferredoxin oxidoreductase and related 2-oxoacid: ferredoxin oxidoreductases (PTH_0056-0059, PTH_1204-1207, PTH_2845 are also genes related to this function, which are not shown for their low abundances and for simplicity, see Supplementary Dataset Pt1 for more details). Enzymes catalyzing each step in *M. conradii* include the following: Ech, energy-converting hydrogenase; Frh, F_420_-reducing hydrogenase; Mvh, F_420_-non-reducing hydrogenase (1 & 2); Fdh, F_420_-reducing formate dehydrogenase; Fwd, CHO-MFR dehydrogenase (Tungsten containing); Fmd, CHO-MFR dehydrogenase (Molybdate containing); Ftr, CHO-MFR: H_4_MPT formyltransferase; Mch, methenyl-H_4_MPT cyclohydrolase (1 & 2); Mtd, methylene-H_4_MPT dehydrogenase; Mer, methylene-H_4_MPT reductase; Mtr, methyl-H_4_MPT: CoM methyltransferase; Mcr, methyl-CoM reductase; Hdr, CoM-S-S-CoB heterodisulfide reductase (1 & 2); ΔμNa^+^, electrochemical sodium ion potential; Drh, putative disulfide reducing hydrogenase complex; Atp, A-type ATP synthase (1 & 2); AtpI, V-type ATP synthase (1 & 2); Acs, acetyl-CoA synthetases/AMP-(fatty) acid ligase (1 & 2); Acd, acetyl-CoA synthetase; Ppa, membrane-bound H^+^-translocating inorganic pyrophosphatase; Por, pyruvate: ferredoxin oxidoreductase (1 & 2); Pdh, pyruvate dehydrogenase; Mtc_1230 and Mtc_1231, putative aconitase subunit (2 & 1); Icd, Isocitrate dehydrogenase; Fum, Fumarate hydratase; Mtc_1389, Citrate synthase; Gln, Glutamine synthetase (1 & 2); GltB, NADPH dependent glutamate synthase (1, 2 & 3); GluQ, ABC-type polar amino acid transport system; Nif, Nitrogenase (1 & 2); Amt, ammonium transporter (1 & 2) (see Supplementary Dataset Mc1 for more details). Abbreviation of chemical compounds: F_420_, coenzyme F_420_ (F_420_H_2_, reduced F_420_); H_4_MPT, tetrahydromethanopterin; MFR, methanofuran; Fd_red_/Fd_ox_, reduced or oxidized ferredoxin; 2-OG, 2-Oxoglutrate; GLU, glutamate; GLN, glutamine; AA, amino acid; MQ, Menaquinone; here was used to simplify the Menaquinone cycle.

The large *mmc* gene cluster (PTH_1356-1369) in *P. thermopropionicum* consists of coding genes for propionyl-CoA: oxaloacetate transcarboxylase (Pot), methylmalonyl-CoA epimerase (Mce), methylmalonyl-CoA mutase (Mcm), succinyl-CoA synthase (Scs), fumarase (Fht), malate dehydrogenase (Mdh), and pyruvate: ferredoxin oxidoreductase (Por). The *mmc* cluster was upregulated more than 4-fold in coculture (Figure 2 and Supplementary Dataset Pt2). The other three key enzymes of the MMC pathway include acyl CoA: acetate/3-ketoacid CoA transferase (Pct), succinate dehydrogenase (Sdh), and acetyl-CoA synthetase (Acs) (Supplementary Dataset Pt2). The genes coding for one Sdh (Sdh I) were upregulated similarly as the *mmc* cluster, while the expression of the second Sdh (Sdh II) was one order of magnitude lower and did not show much response. The coding genes for the two Pct (PTH_2042-2043 and PTH_2044-2045) were upregulated more than 16-fold (L2fc > 4) and the Acs coding gene (PTH_2131) was upregulated to a greater extent (L2fc > 7). For the conversion of pyruvate to acetyl-CoA, the gene coding for pyruvate formate lyase (Pfl) was also upregulated, but the total transcript abundance was much lower than Por coding genes within the *mmc* cluster. Other enzymes related to propionate oxidation, like oxaloacetate decarboxylase (Odc) showed low abundances and the gene coding for malic enzyme (Sfc) was downregulated (Figure 2 and Supplementary Dataset Pt2).

*P*. *thermopropionicum* contains four hydrogenases, i.e., one membrane bound NiFe hydrogenase (NiFe-H_2_ase), one membrane-bound Fe-hydrogenase (Fe-H_2_ase I) and two cytoplasmic Fe-only hydrogenases (Fe-H_2_ases II and III); and two formate dehydrogenases, i.e., a membrane bound formate dehydrogenases (Fdh I) and a cytoplasmic one (Fdh II) (Kosaka et al., 2008). The genes coding for Fe-H_2_ase III (PTH_2010-2012) and Fdh II (PTH_2645-2649) were significantly upregulated (L2fc > 1.2 for Fe-H_2_ase III and L2fc > 4.2 for Fdh II, respectively) (Figure 2, Table 2, and Supplementary Dataset Pt2). The membrane bound Fdh I did not show significant upregulation, while the other three hydrogenases showed low transcript abundances and were moderately downregulated (Figure 2 and Supplementary Dataset Pt2). Multiple sets of putative gene clusters are present in *P*. *thermopropionicum* coding for electron transfer flavoproteins (ETF) that are homologous to the Fix complex in nitrogen-fixing microorganisms. This complex comprises two subunits of the electron transfer flavoprotein (FixA and FixB), one ferredoxin-like protein (FixX), and one membrane-bound ETF-menaquinone oxidoreductase (FixC). Most of the genes of the Fix system were significantly upregulated in coculture (Figure 2, Table 2, and Supplementary Dataset Pt2).

**Table 2:** Transcripts abundance and differential expression of genes coding for putative flavin-based confurcation and bifurcation complexes in *P. thermopropionicum* and *M. conradii*.

**Enzyme**	**Gene ID**	**Gene**	**L2fc^a^**	**L2mFPKM**		**Function**
				**Monoculture**	**Coculture**	
***P. thermopropionicum***						
Fe-hydrogenase (Fe-H_2_ase III)	PTH_2010		1.49	12.05	13.51	Hypothetical hydrogenase subunit
	PTH_2011	*nuoF*	1.19	11.62	12.79	NADH: ubiquinone oxidoreductase, NADH-binding 51 kD subunit
	PTH_2012	*nuoE*	1.68	11.23	12.88	NADH: ubiquinone oxidoreductase, 24 kD subunit
Formate dehydrogenase (Fdh II)	PTH_2645		4.26	7.20	11.53	Hypothetical formate dehydrogenase
	PTH_2646		4.42	7.06	11.57	Hypothetical formate dehydrogenase
	PTH_2647		4.45	7.00	11.53	Hypothetical membrane protein
	PTH_2648	*nuoF*	4.59	6.90	11.56	NADH: ubiquinone oxidoreductase, NADH-binding 51 kD subunit
	PTH_2649	*nuoE*	4.70	5.75	10.54	NADH: ubiquinone oxidoreductase 24 kD subunit
Putative Fix complex I^b^	PTH_0016	*fixA*	1.57	1.61	3.10	Electron transfer flavoprotein, beta subunit
	PTH_0017	*fixB*	1.76	3.97	5.71	Electron transfer flavoprotein, alpha subunit
	PTH_0018	*fixC*	4.55	4.61	9.21	Dehydrogenases
	PTH_0019	*fixX*	2.06	4.40	6.45	Ferredoxin-like protein
Putative Fix complex II^b^	PTH_0597	*fixB*	3.04	1.03	4.42	Electron transfer flavoprotein, alpha subunit
	PTH_0598	*fixC*	2.33	1.68	4.15	Dehydrogenases
	PTH_0599	*fixX*	2.47	2.15	4.79	Ferredoxin-like protein
	PTH_0600	*fixA*	2.44	3.09	5.71	Electron transfer flavoprotein beta subunit
Putative Fix complex III^b^	PTH_1765^c^	*fixX*	n.a.	*n*.*a*.	*n*.*a*.	Ferredoxin-like protein
	PTH_1766	*fixC*	2.26	1.26	3.52	Dehydrogenases
	PTH_1767	*fixB*	1.70	1.39	3.10	Electron transfer flavoprotein, alpha subunit
	PTH_1768	*fixA*	2.35	0.69	3.09	Electron transfer flavoprotein, beta subunit
***M. conradii***						
Fwd-Mvh-Hdr complex^d^	Mtc_2468	*fwdC*	0.81	11.70	12.08	Tungsten-containing formylmethanofuran dehydrogenase, subunit C
	Mtc_2469	*fwdA*	1.31	11.91	12.79	Tungsten-containing formylmethanofuran dehydrogenase, subunit A
	Mtc_2470	*fwdB*	1.02	11.25	11.84	Tungsten-containing formylmethanofuran dehydrogenase subunit B
	Mtc_2471	*fwdD*	0.89	11.29	11.74	Tungsten-containing formylmethanofuran dehydrogenase subunit D
	Mtc_2476	*fwdF*	0.59	10.17	10.32	Tungsten-containing formylmethanofuran dehydrogenase, subunit F
	Mtc_2477	*fwdG*	0.08	9.36	8.99	Tungsten-containing formylmethanofuran dehydrogenase subunit G
	Mtc_2472	*mvhD-2*	0.95	11.58	12.09	F_420_-non-reducing hydrogenase subunit D
	Mtc_2473	*hdrA-2*	0.85	11.16	11.56	CoB-S-S-CoM heterodisulfide reductase subunit A
	Mtc_2474	*hdrB-2*	0.55	10.87	10.98	CoB-S-S-CoM heterodisulfide reductase, subunit B
	Mtc_2475	*hdrC-2*	0.75	11.05	11.35	CoB-S-S-CoM heterodisulfide reductase, subunit C
Formate dehydrogenase (Fdh)^e^	Mtc_2124	*fdhB*	2.81	10.23	12.60	F_420_-reducing formate dehydrogenase, beta subunit
	Mtc_2125	*fdhA*	2.76	10.24	12.55	F_420_-reducing formate dehydrogenase, alpha subunit

*L2fc, log_2_-fold change value; L2mFPKM, log_2_ value of mean FPKM (with 4 replicates); n.a., not applicable*.

a *Differential expression tested by DESeq2 based on raw read counts. Only genes with absolute L2fc ≥ 1.0 and false discovery rate (FDR) < 0.05 were considered to be significantly regulated. Since genes listed here all with FDR < 0.05 except PTH_1765 (See below), FDR are not shown (See Supplementary Datasets Pt2, Mc2 for details)*.

b *For the Fix complex encoding genes, only putative gene clusters with all four fix genes (fixA, fixB, fixC, and fixX) are shown (See Supplementary Dataset Pt2 for more details)*.

c *The transcripts of PTH_1765 is not detected under current sequencing depth*.

d *Shown is the large transcript unit which codes for Fwd, Hdr2, and the subunit D of Mvh2. Proteins may eventually form Fwd-Hdr-Mvh complex for electron bifurcation (See also Figure 2, Figure S7, and Supplementary Dataset Mc2)*.

e *Genes coding for Fdh in M. conradii do not form transcript cluster with Hdr coding genes. But Fdh-Hdr complex is likely to form for electron bifurcation in methanogens according to Costa et al. (2010)*.

*P. thermopropionicum* uses intermediates of the MMC pathway, like 2-oxaloacetate, succinyl-CoA and pyruvate as building bricks for biosynthesis. The transcription of the related genes was generally downregulated in coculture (Figure 2). The transcription of genes for enzymes catalyzing early steps of the glycolysis pathway were also downregulated (Figure S5). *P. thermopropionicum* relies on the assimilation of ammonium and amino acids as nitrogen sources. Notably, the ammonium permease encoding gene (*amtB*) showed very low transcription (L2mFPKM~ = 2). While several ABC-type branched-chain amino acid transport systems were actively transcribed and upregulated (e.g., PTH_0073-0077 and PTH_0458-0462) in coculture (Figure 2 and Supplementary Dataset Pt3). Glutamate synthase coding genes were downregulated, though the expression of the glutamate dehydrogenase gene (*gdhA*) with low abundance apparently increased in coculture (Figure 2 and Supplementary Dataset Pt3). Four of glutamine synthetase encoding genes (*glnA*) were downregulated. The transcription of other amino acids synthetic pathways were downregulated in most cases (Figure S6).

### Metabolic shifts in *M. conradii*

The methanogenesis pathway dominated the transcriptomes of *M*. *conradii* (Supplementary Dataset Mc2). The methyl-CoM reductase (Mcr) coding genes showed the highest transcription followed by methylene-tetrahydromethanopterin (H_4_MPT) dehydrogenase (Mtd), methylene-H_4_MPT reductase (Mer) and methyl-H_4_MPT-CoM methyltranferase (Mtr). The Mcr and Mer coding genes were significantly upregulated. The genes coding for formylmethanofuran dehydrogenase (Fwd), heterodisulde reductase (Hdr) and F_420_-non-reducing hydrogenase (Mvh) form a large gene cluster (Mtc_2468-2479) (Liu et al., 2014; Lyu and Lu, 2015). This cluster was moderately to significantly upregulated (Figure 2, Table 2, and Supplementary Dataset Mc2). The genes coding for formyl-methanofuran-H_4_MPT formyltransferase (Ftr) and methenyl-H_4_MPT cyclohydrolase (Mch) were not affected.

The *M. conradii* genome encodes four types of canonical hydrogenases (Frh, Ech, Mvh1, Mvh2), plus one novel hydrogenase (Drh) that is not found in other methanogens and one F_420_-reducing formate dehydrogenase (Fdh). Apart from Mvh mentioned above the putative membrane-associated novel hydrogenase (Drh) was upregulated while the transcription of F_420_-reducing hydrogenase (Frh) and energy-converting hydrogenase (Ech) did not show an explicit response. Conversely, the transcription of Fdh and formate transporter coding genes were upregulated over 6-fold (L2fc > 2.7) in coculture (Figure 2, Table 2, and Supplementary Dataset Mc2). For ATP synthesis, two of four ATP synthases (Atp1 and AtpI1) showed high transcript abundances but no response to coculture conditions, while the other two (Atp2 and AtpI2) with low abundances were significantly upregulated (Figure 2 and Supplementary Dataset Mc2).

*M. conradii* employs the AMP-forming acetyl-CoA synthetase (Acs) and pyruvate: ferredoxin oxidoreductase (Por) for acetate assimilation and pyruvate conversion. Acs1 and Acs2 were downregulated 3- to 16-fold in coculture (Figure 2 and Supplementary Dataset Mc3). The transcription of inorganic pyrophosphatase (Ppa) coding gene (*ppa*) and Por coding genes also declined but not significantly. One ADP-forming acetyl-CoA synthetase (Acd) coding gene was significantly downregulated. The transcript abundances of pyruvate dehydrogenase (Pdh) coding genes were far lower compared with other carbon assimilation genes. Manual curation revealed that *Methanocella* strains contained citrate synthase and aconitase, indicating the presence of a partial oxidative TCA cycle (Lyu and Lu, 2015). Some of aconitase (Mtc_1230-1231) and isocitrate dehydrogenase (Icd) coding genes were downregulated (Figure 2 and Supplementary Dataset Mc3). A novel citrate synthase encoding gene (Mtc_1389) was actively transcribed but remained unaffected (Figure 2 and Supplementary Dataset Mc3).

Most of the genes involved in nitrogen assimilation system in *M. conradii* were repressed in coculture (Figure 2 and Supplementary Dataset Mc4). The transcription of genes coding for ammonium transporter (Amt) and ABC type amino acid transporter (GluQ) was downregulated 3-fold in coculture. *M. conradii* relies on glutamate and glutamine system for ammonium assimilation. Genes coding for glutamate synthase (Glt) and glutamine synthetase (Gln) were downregulated up to 100-fold in coculture (Figure 2 and Supplementary Dataset Mc4). Although ammonium was present in the culture medium, the transcripts of nitrogenase coding genes were detected. Their transcription however was either unaffected (*nifH1*) or downregulated (*nifH2DKENO*) in coculture (Figure 2 and Supplementary Dataset Mc4).

### Genes for signal transduction system and flagellum

*P. thermopropionicum* and *M. conradii* contain 218 and 101 genes, respectively, coding for proteins related to signal transduction systems (MiST 2.2, http://mistdb.com/). In *P. thermopropionicum*, the transcription of 50 one-component system genes (29 up and 21 down) and 28 two-component system genes (23 up and 5 down) showed explicit shifts in coculture (Supplementary Dataset Pt4). Some of these genes were among those which were most differentially expressed. For example, a gene coding for the response regulator OmpR (PTH_1670) was upregulated 88-fold (L2fc = 6.47) in coculture compared to monoculture (Supplementary Dataset Pt4). In addition, the transcription of two genes (*fliC* and *fliD*) coding for flagella were upregulated significantly in coculture (Figure 2 and Supplementary Dataset Pt5). In *M. conradii*, the expression of 24 one-component system genes (13 up and 11 down) and 16 two-component system genes (11 up and 5 down) changed significantly in coculture (Supplementary Dataset Mc5).

### The effects of sodium 2-bromoethanesulphonate (2-BES), H_2_, and formate on coculture

**Figure 3:**
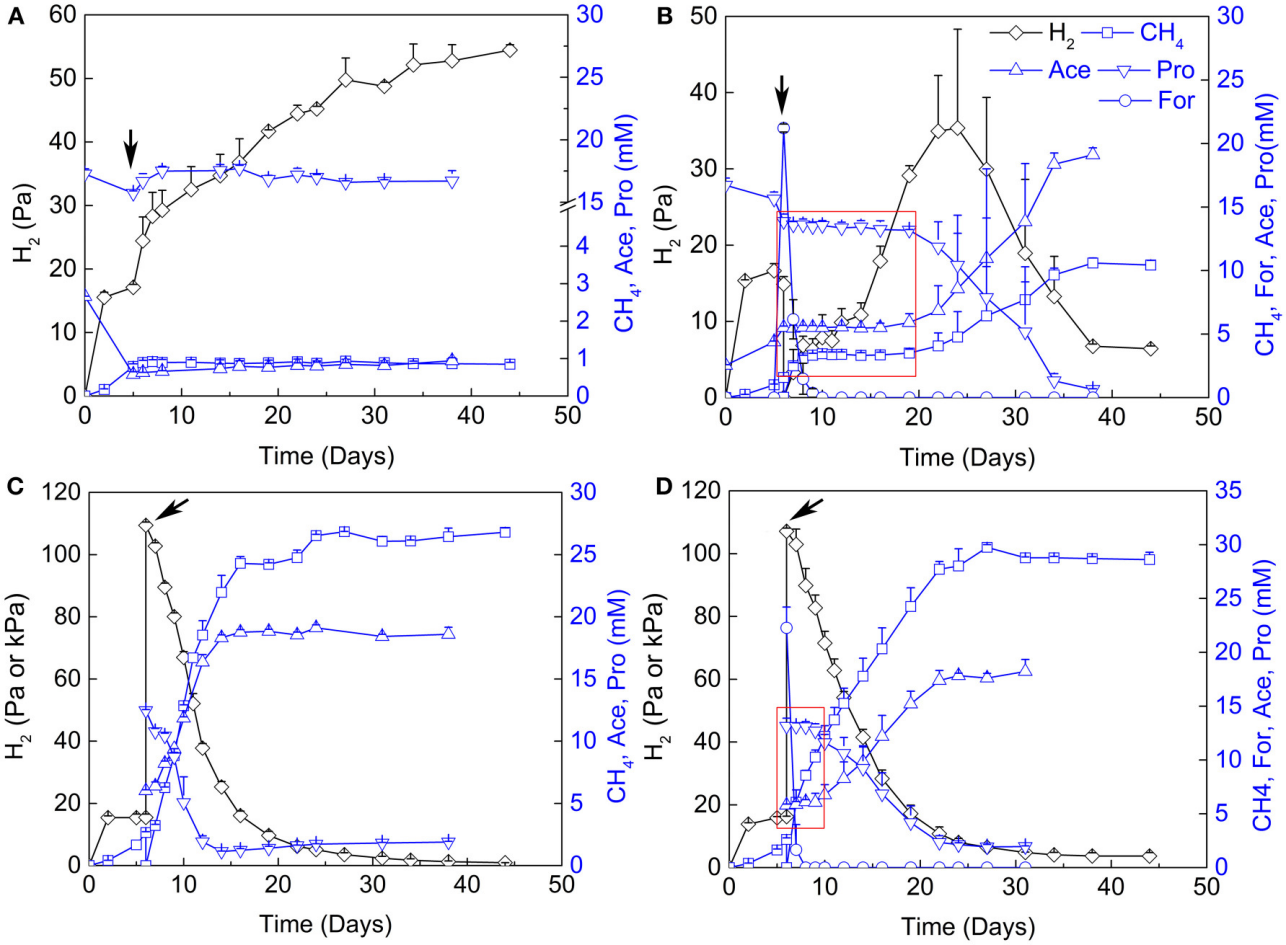
Effects of sodium 2-bromoethanesulphonate (2-BES), H_2_ and formate on syntrophic cocultures. **(A)** addition of 5 mM 2-BES, **(B)** addition of 20 mM formate and reflushed and filled the headspace with 170 kPa N_2_/CO_2_ (80/20, v/v), **(C)** reflushed and filled the headspace with 170 kPa H_2_/CO_2_ (80/20, v/v) and **(D)** addition of 20 mM formate and reflushed and filled the headspace with 170 kPa H_2_/CO_2_ (80/20, v/v). Arrows indicate time points for treatments setup, for **(A)** the 5th day of cultivation, and for **(B–D)**, the 6th day of cultivation. Pa and kPa were used as the unit for H_2_ partial pressures before and after treatments setup, respectively, in **(C,D)**. Time windows with the inhibition effects of formate are emphasized by red rectangle in **(B,D)**. Ace, acetate; Pro, propionate; For, formate.

The contribution of H_2_ vs. formate for interspecies electron transfer remains elusive (de Bok et al., 2004; Schink et al., 2017). Examination of the inhibitory effects of H_2_ and formate has been shown to be an effective way in discerning their contribution (Ahring and Westermann, 1988; Dong et al., 1994). Therefore, 2-BES (5 mM), H_2_/CO_2_ (80/20, v/v, 170 kPa) and formate (20 mM) were added separately or in combination after the initiation of propionate oxidation and CH_4_ production (Figure 3). Methane production and propionate oxidation were completely inhibited after the addition of 2-BES. Hydrogen accumulated to about 55 Pa in the end (Figure 3A), but formate was below the detection limit (~50 μM). The addition of formate halted propionate oxidation and CH_4_ production immediately (Figure 3B). But the added formate was consumed (to CH_4_) within 4 days and the oxidation of propionate resumed 10 days after formate consumption. Hydrogen accumulated to 45 Pa at day 25 and then leveled off. Notably, the addition of H_2_/CO_2_ (80/20, v/v) did not significantly affect propionate oxidation (Figure 3C). Methane production, propionate oxidation, acetate accumulation and H_2_ utilization occurred concomitantly. Addition of both formate and H_2_/CO_2_ (80/20, v/v) inhibited the oxidation of propionate for about 4 days (Figure 3D). In this experiment, formate was converted to CH_4_ within 1 day after addition of formate and the resumption of propionate oxidation was faster than in the treatment with formate addition alone.

## Discussion

### Catabolism vs. anabolism shifts in *P. thermopropionicum* and *M. conradii*

A large proportion of genes in *P. thermopropionicum* and *M. conradii* were differentially transcribed in coculture compared to monoculture. Distinct transcriptome profiles revealed by PCA ordination showed that global metabolism responses occurred in both *P. thermopropionicum* and *M. conradii* under syntrophic coculture condition (Figure 1). It is worth noting that conditions in monoculture are not identical to those in coculture, which can hardly be avoided, since the different microbial communities affect the conditions. The factors (e.g., H_2_ partial pressure, inter-species interactions, the type and concentration of substrate) that may be important for gene expression are to some extent the result of the different organismic compositions, and should be carefully evaluated. However, comparison of gene expression between monoculture and coculture is certainly warranted.

Genes coding for the entire MMC pathway were significantly upregulated in coculture. Although bypaths are present for the conversion of malate to pyruvate (Figure 2), the transcript abundances indicated that *P. thermopropionicum* used malate dehydrogenase and propionyl-CoA: oxaloacetate transcarboxylase instead of malic enzyme and oxaloacetate decarboxylase, consistent with a previous report (Kato et al., 2009). Succinate dehydrogenase (Sdh) catalizes the thermodynamically most endergonic step in propionate oxidation (Schink, 1997). Due to the low redox potential of the electron acceptor, the oxidation of succinate to fumarate must involve reverse electron transport that is probably driven by proton motive force (PMF) (Müller et al., 2010; Schink et al., 2017). PMF is likely formed by ATPase. Unlike the MMC pathway, transcription of the ATPase encoding gene in *P. thermopropionicum* did not significantly respond to coculture conditions. Our results are consistent with previous proteomic analyses, in which subunits of ATPase were major spots in the gel but showed less response to syntrophic propionate coculture conditions than the protein spots of the MMC pathway catalyzing enzymes (Kosaka et al., 2006). We assume that the insignificant response of ATPase implies a strategy for avoiding the waste of PMF or the slowdown of growth under coculture condition. The enzyme Pct is proposed to catabolize the formation of propionyl-CoA from propionate by coupling the conversion of acetyl-CoA to acetate, i.e., the first and the last step of the MMC pathway, respectively (Kosaka et al., 2006, 2008) (Figure 2). The enzyme Acs has been proposed to also catabolize the hydrolysis of acetyl-CoA, coupled to ATP formation (the last step of MMC pathway) (Kosaka et al., 2006) (Figure 2). Notably, the expression of Pct and Acs genes was upregulated to greater extents than that of the *mmc* cluster, especially the Acs gene showing the largest upregulation. High pyrophosphate levels and a high AMP-to-ATP ratio in syntrophic bacterium *Syntrophus aciditrophicus* cells support the operation of Acs in the direction of acetate and ATP forming (James et al., 2016). If similar high AMP-to-ATP ratio was maintained by *P. thermopropionicum*, then the activity of Acs together with succinyl-CoA synthase (Scs) probably supported CoA recycling and ATP generation via substrate-level phosphorylation. Otherwise, Acs would operate in the direction of acetate-CoA formation, securing the pool of acetyl-CoA. The upregulation of the first and last steps could help securing the activity of the MMC pathway and the conservation of energy under coculture condition. In contrast, most of the genes involved in biosynthetic processes were downregulated (Figure 2 and Figures S5, S6). This downregulation coincided with a reduced growth rate of *P. thermopropionicum* in coculture (Figure S1).

Similar to *P. thermopropionicum, M. conradii* tended to upregulate catabolism in coculture compared to monoculture. Genes coding for Mer and Mcr were not only upregulated but exhibited the highest transcript abundances (Figure 2 and Supplementary Dataset Mc2). Notably, F_420_-reducing formate dehydrogenase (Fdh) but not F_420_-reducing hydrogenase (Frh) was significantly upregulated (Figure 2, Table 2, and Supplementary Dataset Mc2). This result possibly indicates that formate plays an important role (see more discussion below). The concomitant upregulation of Fdh and Mer activity indicates that larger proportions of electron flow were channeled into methanogenesis pathway in coculture compared to monoculture. Another route for electron flow is the putative Fwd-Mvh-Hdr complex, which was proposed to connect the first and last steps of the methanogenesis pathway and mediates electron bifurcation (Liu et al., 2014). The genes coding for this route were significantly upregulated (Figure 2, Table 2, and Supplementary Dataset Mc2). In addition, genes coding for two ATPases (AtpI2 and Atp2) were upregulated. ATPases are proposed to produce ATP using the Na^+^/H^+^ motive force generated from Mtr activity (Lyu and Lu, 2015). Thus, under coculture condition *M. conradii* enhanced several key steps for methanogenic energy formation, i.e., the methyl CoM reductase (Mcr), two routes of electron flow and ATPase. By contrast, most of the genes associated with carbon assimilation and nitrogen metabolisms were significantly downregulated in coculture (Figure 2, Figures S7, S8).

Taken together, both *P. thermopropionicum* and *M. conradii* enhanced catabolism but decreased anabolism under syntrophic conditions. It is believed that growth rate affects gene expression in both bacteria and archaea. Therefore, the responses observed may include the effect of growth rate in addition to syntrophy. Because the growth rate of both organisms was lower under coculture than monoculture conditions, this kind of metabolic shift probably reflects an universal strategy for microbes to survive environmental stress or harsh conditions (Goodchild et al., 2005; Kato et al., 2008, 2009; Enoki et al., 2011).

### Flavin-based electron bifurcation/confurcation

Under conditions of thermodynamic limitation the ability to maximize energy conservation should be an advantage for the growth of syntrophic partners (Sieber et al., 2012). Flavin-based electron bifurcation/confurcation that couples exergonic with endergonic redox reactions has been proposed as the third form of energy conservation in microbial life (Herrmann et al., 2008; Buckel and Thauer, 2018). This process seems to be widespread in anaerobic microorganisms and offers a solution for their energetic dilemma (Buckel and Thauer, 2013; Peters et al., 2016). Three putative electron bifurcation/confurcation systems are found in *P. thermopropionicum*. First, the cytoplasmic Fe-H_2_ase III contains NADH dehydrogenase and shows sequence homology with the electron confurcating Hyd complex of *Thermotoga maritima*. Therefore, it could be a putative site for electron confurcation (Sieber et al., 2010, 2012). Second, the genes coding for catalytic subunits of Fdh II are associated with genes coding for NADH-quinone oxidoreductase, thus suggesting that this enzyme could catalyze electron confurcation. Thus, H_2_ and formate could be produced by coupling the reoxidation of NADH with the reduction of ferredoxin (Fd_red_) by Fe-H_2_ase III and Fdh II, respectively. Notably, both Fe-H_2_ase III and Fdh II were actively transcribed and significantly upregulated (Figure 2 and Table 2). Third, the Fix complex has been shown to perform electron bifurcation in the N_2_-fixing *Azotobacter vinelandii* for the production of Fd_red_ from NADH (Ledbetter et al., 2017). Most of Fix encoding genes were markedly upregulated in coculture (Figure 2 and Table 2). The Fix system was also found in the genome of butyrate-oxidizing *S. wolfei* and was proposed to produce Fd_red_, which is needed for the biosynthesis of hydrogen and/or formate from NADH (Sieber et al., 2010). However, the necessity of electron bifurcation/confurcation in *S. wolfei* has been questioned recently, since the beta oxidation pathway in butyrate degradation does not involve Fd_red_ production, and since the H_2_ partial pressure under syntrophic conditions was sufficiently low for direct reoxidation of NADH (Losey et al., 2017). However, for propionate oxidation in *P. thermopropionicum* where pyruvate: ferredoxin oxidoreductase coding genes were actively transcribed and upregulated under coculture condition (Figure 2), production of Fd_red_ from pyruvate oxidation and coupling of NADH oxidation with Fd_red_ reduction is quite liekely. The activity of the Fix enzyme may play an anaplerotic role in maintaining NADH and Fd_red_ equilibrium during propionate oxidation and/or supporting the needs of biosynthesis. Further investigations are necessary to elucidate why genes for all putative electron bifurcation/confurcation systems (Fe-H_2_ase III, Fdh II, and Fix) are upregulated in *P. thermopropionicum* during syntrophic growth.

In hydrogenotrophic methanogens, Mvh and Hdr proteins form a complex that performs flavin-based electron bifurcation to simultaneously reduce CoM-S-S-CoB heterodisulfide and ferredoxin, which is required for the reduction of CO_2_ to formylmethanofuran (Kaster et al., 2011; Wagner et al., 2017). This coupling mechanism together with the anaplerotic reaction catalyzed by energy converting hydrogenase (Lie et al., 2012) completes the reduction of CO_2_ to methane in a full cycle (i.e., the Wolfe cycle), with ferredoxin coupling the first and last steps (Kaster et al., 2011; Thauer, 2012). The presence of a multienzyme supercomplex in *Methanococcus maripaludis*, comprising not only Hdr and Vhu (equivalent to Mvh in *M. conradii*) but also Fwd and Fdh, indicates that ferredoxin pools are probably compartmentalized and formate dehydrogenase is directly involved in this process (Costa et al., 2010; Thauer, 2012). We found that the transcription of Fdh coding genes was upregulated under coculture condition to a significantly greater extent than that of Mvh genes. Therefore, it is probable that formate donates electrons to the electron bifurcation Mvh-Hdr complex via Fdh in *M. conradii*, similarly as in *M. maripaludis*. The *M. conradii* genome predicts the presence of a large transcript unit comprising genes coding for Hdr, Fwd and a subunit of Mvh (Lyu and Lu, 2015). The transcriptome analysis disclosed that this cluster was transcribed indeed as a single unit in *M. conradii* (Figure S9 and Table S5) and was significantly upregulated in coculture (Figure 2 and Table 2). Comparative genomics indicates that the large transcript unit comprising Hdr and Fwd is only present in *Methanocella* strains among all methanogens known to date (Lyu and Lu, 2015). This feature may make *Methanocella* unique for constituting the Wolfe cycle and assembling its bifurcating multienzyme complex, and thus optimize energy conservation. Optimized energy conservation in *Methanocella* may be the reason for the extraordinary adaption to H_2_-limiting conditions (Lu et al., 2005) and the ability to outcompete other methanogens for forming a syntrophic relationship with fatty acid oxidizing bacteria (Sakai et al., 2007; Liu P. et al., 2011; Rui et al., 2011; Gan et al., 2012).

### Formate as a mediator for interspecies electron transfer

It has been assumed that *P. thermopropionicum* is not likely to use formate as electron mediator for syntrophic growth with methanogens (Imachi et al., 2002). Indeed, *M. conradii* in pure culture cannot grow on formate alone (Lü and Lu, 2012). In the present experiment, however, we found that the cytoplasmic Fdh II in *P. thermopropionicum* was upregulated to a significantly greater extent than any of its cytoplasmic Fe-H_2_ases. Similarly, the membrane bound Fdh I was also upregulated (albeit not significantly) while its H_2_ase counterparts appeared to be downregulated under syntrophic condition. Coincidently, Fdh in *M. conradii* was upregulated in coculture to a greater extent than both F_420_-reducing (Frh) and F_420_-non-reducing hydrogenases (Mvh). Our experiments showed that the addition of formate to an active coculture suppressed the syntrophic activity immediately (Figure 3B). It took a substantial time lag before the coculture resumed its activity even though formate was already consumed. On the contrary, the addition of H_2_ instead of formate resulted in very little effect (Figure 3C). Notably, the addition of H_2_ together with formate caused inhibition but not as significant as formate alone. H_2_ did not result in an additional negative effect but instead accelerated the consumption of formate by *M. conradii* and pushed the recovery of syntrophic activity (Figure 3D). Taken together, our study suggests that formate played an important role in interspecies electron transfer between *P. thermopropionicum* and *M. conradii*. This conclusion is supported by our previous observation that the expression of a gene coding for the alpha subunit of Fdh in *M. conradii* is upregulated under syntrophic conditions and *M. conradii*, albeit not using formate alone, used formate in the presence of H_2_ (Liu et al., 2014). A previous study showed that H_2_ was essential for *M. maripaludis* to grow on formate where H_2_ was either produced internally (from formate) or supplied externally (Lie et al., 2012). This essentiality was considered to be associated with the need for the activity of a membrane bound energy-converting hydrogenase (Eha). Whether a similar mechanism works in *M. conradii* deserves further investigations.

Despite the importance of formate, a role of H_2_ for interspecies electron transfer cannot be ruled out. Genes coding for hydrogenases in both *P. thermopropionicum* (Fe-H_2_ase III in particular) and *M. conradii* (Mvh and Frh) were actively transcribed. Production and consumption of H_2_ occurred simultaneously during syntrophic growth (Figure 3). Syntrophic growth was not inhibited by high partial pressure of H_2_ might be due to diffusion limits under static culture conditions. We assume that optimal syntrophic growth requires both formate and H_2_ for interspecies electron transfer in coculture. The necessity of formate together with H_2_ was also demonstrated in *Syntrophobacter fumaroxidans* (Dong et al., 1994; Dong and Stams, 1995; Worm et al., 2011a,b; Sedano-Núñez et al., 2018).

### Other possible mechanisms

Additional mechanisms, such as signal transduction by flagella or amino acid transport, may be important for syntrophic interactions between *P. thermopropionicum* and *M. conradii*. Flagella are considered allowing syntrophic bacteria to effectively interact with their methanogen partners (Ishii et al., 2006; Krumholz et al., 2015). The flagellar cap protein (FliD) apparently induces signal transduction between *P. thermopropionicum* and *M. thermautotrophicus* (Shimoyama et al., 2009). Our transcriptome analysis showed that transcription of flagellum biosynthesis genes (especially FliD coding gene) was markedly enhanced in coculture suggesting its implication for syntrophic growth.

Transcriptome analysis also showed that the abundance of ammonium transporters was exceptionally low while amino acid transporter coding genes were upregulated, indicating that *P. thermopropionicum* may rely on amino acid uptake. This is consistent with proteomic analyses in which a subunit of the branched-chain amino acid ABC transporter was the major soluble protein under coculture conditions (Kosaka et al., 2006). The importance of amino acid transport for syntrophic conditions is also in line with the previous observation that *P. thermopropionicum* can grow syntrophically with *M. thermautotrophicus* in the absence of yeast extract but requires yeast extract in monoculture (Kato et al., 2009). Transfer of amino acids has also been proposed for a syntrophic coculture of *Desulfovibrio vulgaris* and *M. maripaludis* (Walker et al., 2012). Exchange of amino acids may be an evolutionarily optimizing strategy to reduce the biosynthetic burden while promoting the cooperative interactions between different bacteria in the microbiome (Mee et al., 2014). We hypothesize that amino acid transfer from *M. conradii* to *P. thermopropionicum* compensates the low free energy gain during propionate degradation.

## Conclusions

Our coculture experiments and transcriptome analyses disclosed several aspects of coordinated metabolic shifts in both *P. thermopropionicum* and *M. conradii* to cope with syntrophic growth. Firstly, both organisms tended to decrease energy-consuming biosynthesis pathways and enhance energy-generating catabolic pathways. This strategy is in accordance with the reduced growth rate of both organisms in coculture compared to monoculture. Secondly, genes coding for flavin-based electron bifurcation/confurcation systems were substantially upregulated in *P. thermopropionicum* and *M. conradii* under coculture condition. Fdh-based multienzyme complex might be formed in addition to the Mvh-Hdr complex in *M. conradii* for electron bifurcation. This study shows the importance of electron bifurcation/confurcation for syntrophic partners to maximize their energy conservation. Thirdly, formate was found to act besides H_2_ as an important mediator for interspecies electron transfer between the two syntrophic partners and possibly renders syntrophic propionate oxidation more efficient. Previously studies only revealed the utilization of formate by *M. conradii* in monoculture in the presence of H_2_ and the capacity of *P. thermopropionicum* to produce formate for the recycling of redox mediators (e.g., NADH) during syntrophic propionate oxidation was unclear. The transcriptome analysis also indicated that additional mechanisms like the flagellum mediated signal transduction and amino acids exchange was important for syntrophic growth. Apparently, *P. thermopropionicum* and *M. conradii*, when growing syntrophically, are employing multiple mechanisms for optimizing their cooperation under energy limitation condition. Syntrophic interactions under *in situ* conditions, however, may be even more complicated. Redundant copies of functional genes like multiple cytoplasmic and membrane-bound hydrogenases are present in both *P. thermopropionicum* and *M. conradii* genomes. How these different gene products coordinate and work together under varying environmental conditions shall deserve further investigations.

## Author contributions

PL and YL designed the experiment. PL performed the experiment and analyzed data. PL and YL wrote the main manuscript. PL and YL revised and approved the final version of the manuscript.

### Conflict of interest statement

The authors declare that the research was conducted in the absence of any commercial or financial relationships that could be construed as a potential conflict of interest.

## References

[B1] Ahring B. K. Westermann P. (1988). Product inhibition of butyrate metabolism by acetate and hydrogen in a thermophilic coculture. Appl. Environ. Microbiol. 54, 2393–2397. 16347751 10.1128/aem.54.10.2393-2397.1988 PMC204269

[B2] Anders S. Pyl P. T. Huber W. (2015). HTSeq—a Python framework to work with high-throughput sequencing data. Bioinformatics 31, 166–169. 10.1093/bioinformatics/btu638 25260700 PMC4287950

[B3] Angel R. Claus P. Conrad R. (2012). Methanogenic archaea are globally ubiquitous in aerated soils and become active under wet anoxic conditions. ISME J. 6, 847–862. 10.1038/ismej.2011.141 22071343 PMC3309352

[B4] Angel R. Matthies D. Conrad R. (2011). Activation of methanogenesis in arid biological soil crusts despite the presence of oxygen. PLoS ONE 6:e20453. 10.1371/journal.pone.0020453 21655270 PMC3105065

[B5] Aschenbach K. Conrad R. Reháková K. DoleŽal J. Janatková K. Angel R. (2013). Methanogens at the top of the world: occurrence and potential activity of methanogens in newly deglaciated soils in high-altitude cold deserts in the Western Himalayas. Front. Microbiol. 4:359. 10.3389/fmicb.2013.00359 24348469 PMC3847552

[B6] Buckel W. Thauer R. K. (2013). Energy conservation via electron bifurcating ferredoxin reduction and proton/Na^+^ translocating ferredoxin oxidation. Biochim. Biophys. Acta Bioener. 1827, 94–113. 10.1016/j.bbabio.2012.07.002 22800682

[B7] Buckel W. Thauer R. K. (2018). Flavin-based electron bifurcation, a new mechanism of biological energy coupling. Chem. Rev. 118, 3862–3886. 10.1021/acs.chemrev.7b00707 29561602

[B8] Conrad R. (1999). Contribution of hydrogen to methane production and control of hydrogen concentrations in methanogenic soils and sediments. FEMS Microbiol. Ecol. 28, 193–202. 10.1111/j.1574-6941.1999.tb00575.x

[B9] Conrad R. Erkel C. Liesack W. (2006). Rice Cluster I methanogens, an important group of Archaea producing greenhouse gas in soil. Curr. Opin. Biotechnol. 17, 262–267. 10.1016/j.copbio.2006.04.002 16621512

[B10] Costa K. C. Wong P. M. Wang T. Lie T. J. Dodsworth J. A. Swanson I.. (2010). Protein complexing in a methanogen suggests electron bifurcation and electron delivery from formate to heterodisulfide reductase. Proc. Natl. Acad. Sci. U.S.A. 107, 11050–11055. 10.1073/pnas.1003653107 20534465 PMC2890747

[B11] de Bok F. A. Stams A. J. Dijkema C. Boone D. R. (2001). Pathway of propionate oxidation by a syntrophic culture of *Smithella propionica* and *Methanospirillum hungatei*. Appl. Environ. Microbiol. 67, 1800–1804. 10.1128/AEM.67.4.1800-1804.2001 11282636 PMC92800

[B12] de Bok F. A. M. Plugge C. M. Stams A. J. M. (2004). Interspecies electron transfer in methanogenic propionate degrading consortia. Water. Res. 38, 1368–1375. 10.1016/j.watres.2003.11.028 15016514

[B13] Dong X. Plugge C. M. Stams A. J. M. (1994). Anaerobic degradation of propionate by a mesophilic acetogenic bacterium in coculture and triculture with different methanogens. Appl. Environ. Microbiol. 60, 2834–2838. 16349350 10.1128/aem.60.8.2834-2838.1994 PMC201730

[B14] Dong X. Stams A. J. (1995). Evidence for H_2_ and formate formation during syntrophic butyrate and propionate degradation. Anaerobe 1, 35–39. 10.1016/S1075-9964(95)80405-6 16887505

[B15] Enoki M. Shinzato N. Sato H. Nakamura K. Kamagata Y. (2011). Comparative proteomic analysis of *Methanothermobacter themautotrophicus* ΔH in pure culture and in co-culture with a butyrate-oxidizing bacterium. PLoS ONE 6:e24309. 10.1371/journal.pone.0024309 21904627 PMC3164167

[B16] Gan Y. Qiu Q. Liu P. Rui J. Lu Y. (2012). Syntrophic oxidation of propionate in rice field soil at 15 and 30°C under methanogenic conditions. Appl. Environ. Microbiol. 78, 4923–4932. 10.1128/AEM.00688-12 22582054 PMC3416378

[B17] Giannoukos G. Ciulla D. M. Huang K. Haas B. J. Izard J. Levin J. Z.. (2012). Efficient and robust RNA-Seq process for cultured bacteria and complex community transcriptomes. Genome Biol. 13:R23. 10.1186/gb-2012-13-3-r23 22455878 PMC3439974

[B18] Glissmann K. Conrad R. (2000). Fermentation pattern of methanogenic degradation of rice straw in anoxic paddy soil. FEMS Microbiol. Ecol. 31, 117–126. 10.1111/j.1574-6941.2000.tb00677.x 10640665

[B19] Goodchild A. Raftery M. Saunders N. F. Guilhaus M. Cavicchioli R. (2005). Cold adaptation of the antarctic archaeon, *Methanococcoides burtonii* assessed by proteomics using ICAT. J. Proteome Res. 4, 473–480. 10.1021/pr049760p 15822924

[B20] Hendrickson E. L. Haydock A. K. Moore B. C. Whitman W. B. Leigh J. A. (2007). Functionally distinct genes regulated by hydrogen limitation and growth rate in methanogenic Archaea. Proc. Natl. Acad. Sci. U.S.A. 104, 8930–8934. 10.1073/pnas.0701157104 17502615 PMC1885605

[B21] Herrmann G. Jayamani E. Mai G. Buckel W. (2008). Energy conservation via electron-transferring flavoprotein in anaerobic bacteria. J. Bacteriol. 190, 784–791. 10.1128/JB.01422-07 18039764 PMC2223574

[B22] Hrdlickova R. Toloue M. Tian B. (2017). RNA-Seq methods for transcriptome analysis. Wiley Interdiscip. Rev. RNA 8:e1364. 10.1002/wrna.1364 27198714 PMC5717752

[B23] Imachi H. Sekiguchi Y. Kamagata Y. Hanada S. Ohashi A. Harada H. (2002). *Pelotomaculum thermopropionicum* gen. nov., sp nov., an anaerobic, thermophilic, syntrophic propionate-oxidizing bacterium. Int. J. Syst. Evol. Microbiol. 52, 1729–1735. 10.1099/00207713-52-5-1729 12361280

[B24] Ishii S. Kosaka T. Hori K. Hotta Y. Watanabe K. (2005). Coaggregation facilitates interspecies hydrogen transfer between *Pelotomaculum thermopropionicum* and *Methanothermobacter thermautotrophicus*. Appl. Environ. Microbiol. 71, 7838–7845. 10.1128/AEM.71.12.7838-7845.2005 16332758 PMC1317437

[B25] Ishii S. Kosaka T. Hotta Y. Watanabe K. (2006). Simulating the contribution of coaggregation to interspecies hydrogen fluxes in syntrophic methanogenic consortia. Appl. Environ. Microbiol. 72, 5093–5096. 10.1128/AEM.00333-06 16820513 PMC1489340

[B26] James K. L. Ríos-Hernández L. A. Wofford N. Q. Mouttaki H. Sieber J. R. Sheik C. S.. (2016). Pyrophosphate-dependent ATP formation from Acetyl Coenzyme A in *Syntrophus aciditrophicus*, a new twist on ATP formation. mBio 7:e01208-16. 10.1128/mBio.01208-16 27531911 PMC4992975

[B27] Kaster A. K. Moll J. Parey K. Thauer R. K. (2011). Coupling of ferredoxin and heterodisulfide reduction via electron bifurcation in hydrogenotrophic methanogenic archaea. Proc. Natl. Acad. Sci. U.S.A. 108, 2981–2986. 10.1073/pnas.1016761108 21262829 PMC3041090

[B28] Kato S. Kosaka T. Watanabe K. (2008). Comparative transcriptome analysis of responses of *Methanothermobacter thermautotrophicus* to different environmental stimuli. Environ. Microbiol. 10, 893–905. 10.1111/j.1462-2920.2007.01508.x 18036179

[B29] Kato S. Kosaka T. Watanabe K. (2009). Substrate-dependent transcriptomic shifts in *Pelotomaculum thermopropionicum* grown in syntrophic co-culture with *Methanothermobacter thermautotrophicus*. Microb. Biotechnol. 2, 575–584. 10.1111/j.1751-7915.2009.00102.x 21255290 PMC3815365

[B30] Kosaka T. Kato S. Shimoyama T. Ishii S. Abe T. Watanabe K. (2008). The genome of *Pelotomaculum thermopropionicum* reveals niche-associated evolution in anaerobic microbiota. Genome Res. 18, 442–448. 10.1101/gr.7136508 18218977 PMC2259108

[B31] Kosaka T. Uchiyama T. Ishii S. Enoki M. Imachi H. Kamagata Y.. (2006). Reconstruction and regulation of the central catabolic pathway in the thermophilic propionate-oxidizing syntroph *Pelotomaculum thermopropionicum*. J. Bacteriol. 188, 202–210. 10.1128/JB.188.1.202-210.2006 16352836 PMC1317604

[B32] Krumholz L. R. Bradstock P. Sheik C. S. Diao Y. Gazioglu O. Gorby Y.. (2015). Syntrophic growth of *Desulfovibrio alaskensis* requires genes for H_2_ and formate metabolism as well as those for flagellum and biofilm formation. Appl. Environ. Microbiol. 81, 2339–2348. 10.1128/AEM.03358-14 25616787 PMC4357941

[B33] Lü Z. Lu Y. (2012). Methanocella conradii sp. nov., a thermophilic, obligate hydrogenotrophic methanogen, isolated from Chinese rice field soil. PLoS ONE 7:e35279 10.1371/journal.pone.0035279 PMC3328440 22530002

[B34] Lyu Z. Lu Y. (2015). Comparative genomics of three *Methanocellales* strains reveal novel taxonomic and metabolic features. Environ. Microbiol. Rep. 7, 526–537. 10.1111/1758-2229.12283 25727385

[B35] Ledbetter R. N. Garcia Costas A. M. Lubner C. E. Mulder D. W. Tokmina-Lukaszewska M. Artz J. H.. (2017). The electron bifurcating FixABCX protein complex from *Azotobacter vinelandii*: generation of low-potential reducing equivalents for nitrogenase catalysis. Biochemistry 56, 4177–4190. 10.1021/acs.biochem.7b00389 28704608 PMC7610252

[B36] Levin J. Z. Yassour M. Adiconis X. Nusbaum C. Thompson D. A. Friedman N.. (2010). Comprehensive comparative analysis of strand-specific RNA sequencing methods. Nat. Methods 7, 709–715. 10.1038/nmeth.1491 20711195 PMC3005310

[B37] Li H. Durbin R. (2009). Fast and accurate short read alignment with Burrows–Wheeler transform. Bioinformatics 25, 1754–1760. 10.1093/bioinformatics/btp324 19451168 PMC2705234

[B38] Li H. Handsaker B. Wysoker A. Fennell T. Ruan J. Homer N.. (2009). The sequence alignment/map format and SAMtools. Bioinformatics 25, 2078–2079. 10.1093/bioinformatics/btp352 19505943 PMC2723002

[B39] Lie T. J. Costa K. C. Lupa B. Korpole S. Whitman W. B. Leigh J. A. (2012). Essential anaplerotic role for the energy-converting hydrogenase Eha in hydrogenotrophic methanogenesis. Proc. Natl. Acad. Sci. U.S.A. 109, 15473–15478. 10.1073/pnas.1208779109 22872868 PMC3458328

[B40] Liu F. Conrad R. (2010). *Thermoanaerobacteriaceae* oxidize acetate in methanogenic rice field soil at 50°C. Environ. Microbiol. 12, 2341–2354. 10.1111/j.1462-2920.2010.02289.x 21966924

[B41] Liu H. Wang J. Wang A. Chen J. (2011). Chemical inhibitors of methanogenesis and putative applications. Appl. Microbiol. Biotechnol. 89, 1333–1340. 10.1007/s00253-010-3066-5 21193988

[B42] Liu L. Hu N. Wang B. Chen M. Wang J. Tian Z. (2011). A brief utilization report on the Illumina HiSeq 2000 sequencer. Mycology 2, 169–191. 10.1080/21501203.2011.615871

[B43] Liu P. Qiu Q. Lu Y. (2011). *Syntrophomonadaceae*-affiliated species as active butyrate-utilizing syntrophs in paddy field soil. Appl. Environ. Microbiol. 77, 3884–3887. 10.1128/AEM.00190-11 21460111 PMC3127591

[B44] Liu P. Yang Y. Lü Z. Lu Y. (2014). Response of a paddy soil methanogen to syntrophic growth as revealed by transcriptional analyses. Appl. Environ. Microbiol. 80, 4668–4676. 10.1128/AEM.01259-14 24837392 PMC4148802

[B45] Losey N. A. Mus F. Peters J. W. Le H. M. Mcinerney M. J. (2017). *Syntrophomonas wolfei* uses a NADH-dependent, ferredoxin-independent, [FeFe]-hydrogenase to reoxidize NADH. Appl. Environ. Microbiol. 83, e01335-17. 10.1128/AEM.01335-17 28802265 PMC5626996

[B46] Love M. I. Huber W. Anders S. (2014). Moderated estimation of fold change and dispersion for RNA-Seq data with DESeq2. Genome Biol. 15, 550. 10.1186/s13059-014-0550-8 25516281 PMC4302049

[B47] Lu Y. Lueders T. Friedrich M. W. Conrad R. (2005). Detecting active methanogenic populations on rice roots using stable isotope probing. Environ. Microbiol. 7, 326–336. 10.1111/j.1462-2920.2005.00697.x 15683393

[B48] Lueders T. Pommerenke B. Friedrich M. W. (2004). Stable-isotope probing of microorganisms thriving at thermodynamic limits: syntrophic propionate oxidation in flooded soil. Appl. Environ. Microbiol. 70, 5778–5786. 10.1128/AEM.70.10.5778-5786.2004 15466514 PMC522077

[B49] McGettigan P. A. (2013). Transcriptomics in the RNA-Seq era. Curr. Opin. Chem. Biol. 17, 4–11. 10.1016/j.cbpa.2012.12.008 23290152

[B50] McInerney M. J. Sieber J. R. Gunsalus R. P. (2009). Syntrophy in anaerobic global carbon cycles. Curr. Opin. Biotechnol. 20, 623–632. 10.1016/j.copbio.2009.10.001 19897353 PMC2790021

[B51] McInerney M. J. Struchtemeyer C. G. Sieber J. Mouttaki H. Stams A. J. Schink B.. (2008). Physiology, ecology, phylogeny, and genomics of microorganisms capable of syntrophic metabolism. Ann. N. Y. Acad. Sci. 1125, 58–72. 10.1196/annals.1419.005 18378587

[B52] Mee M. T. Collins J. J. Church G. M. Wang H. H. (2014). Syntrophic exchange in synthetic microbial communities. Proc. Natl. Acad. Sci. U.S.A. 111, E2149–E2156. 10.1073/pnas.1405641111 24778240 PMC4034247

[B53] Müller N. Worm P. Schink B. Stams A. J. Plugge C. M. (2010). Syntrophic butyrate and propionate oxidation processes: from genomes to reaction mechanisms. Environ. Microbiol. Rep. 2, 489–499. 10.1111/j.1758-2229.2010.00147.x 23766220

[B54] Nobu M. K. Narihiro T. Rinke C. Kamagata Y. Tringe S. G. Woyke T.. (2015). Microbial dark matter ecogenomics reveals complex synergistic networks in a methanogenic bioreactor. ISME J. 9, 1710–1722. 10.1038/ismej.2014.256 25615435 PMC4511927

[B55] Nölling J. Reeve J. N. (1997). Growth- and substrate-dependent transcription of the formate dehydrogenase (*fdhCAB*) operon in *Methanobacterium thermoformicicum* Z-245. J. Bacteriol. 179, 899–908. 10.1128/jb.179.3.899-908.1997 9006048 PMC178775

[B56] Parkhomchuk D. Borodina T. Amstislavskiy V. Banaru M. Hallen L. Krobitsch S.. (2009). Transcriptome analysis by strand-specific sequencing of complementary DNA. Nucleic Acids Res. 37, e123. 10.1093/nar/gkp596 19620212 PMC2764448

[B57] Peters J. W. Miller A. F. Jones A. K. King P. W. Adams M. W. (2016). Electron bifurcation. Curr. Opin. Chem. Biol. 31, 146–152. 10.1016/j.cbpa.2016.03.007 27016613

[B58] R Core Team (2016). R: A Language and Environment for Statistical Computing. Vienna: R foundation for statistical computing. Available online at: http://www.r-project.org/

[B59] Rothfuss F. Conrad R. (1993). Thermodynamics of methanogenic intermediary metabolism in littoral sediment of Lake Constance. FEMS Microbiol. Ecol. 12, 265–276. 10.1111/j.1574-6941.1993.tb00039.x

[B60] Rui J. Qiu Q. Lu Y. (2011). Syntrophic acetate oxidation under thermophilic methanogenic condition in Chinese paddy field soil. FEMS Microbiol. Ecol. 77, 264–273. 10.1111/j.1574-6941.2011.01104.x 21470253

[B61] Sakai S. Conrad R. Liesack W. Imachi H. (2010). *Methanocella arvoryzae* sp. nov., a hydrogenotrophic methanogen isolated from rice field soil. Int. J. Syst. Evol. Microbiol. 60, 2918–2923. 10.1099/ijs.0.020883-0 20097796

[B62] Sakai S. Imachi H. Hanada S. Ohashi A. Harada H. Kamagata Y. (2008). *Methanocella paludicola gen*. nov., sp. nov., a methane-producing archaeon, the first isolate of the lineage 'Rice Cluster I', and proposal of the new archaeal order *Methanocellales* ord. nov. Int. J. Syst. Evol. Microbiol. 58, 929–936. 10.1099/ijs.0.65571-0 18398197

[B63] Sakai S. Imachi H. Sekiguchi Y. Ohashi A. Harada H. Kamagata Y. (2007). Isolation of key methanogens for global methane emission from rice paddy fields: a novel isolate affiliated with the clone cluster rice cluster I. Appl. Environ. Microbiol. 73, 4326–4331. 10.1128/AEM.03008-06 17483259 PMC1932770

[B64] Sakai S. Imachi H. Sekiguchi Y. Tseng I.-C. Ohashi A. Harada H.. (2009). Cultivation of methanogens under low-hydrogen conditions by using the coculture method. Appl. Environ. Microbiol. 75, 4892–4896. 10.1128/AEM.02835-08 19465530 PMC2708418

[B65] Schink B. (1997). Energetics of syntrophic cooperation in methanogenic degradation. Microbiol. Mol. Biol. Rev. 61, 262–280. 9184013 10.1128/mmbr.61.2.262-280.1997 PMC232610

[B66] Schink B. Stams A. J. M. (2006). Syntrophism among prokaryotes, in Prokaryotes: A Handbook on the Biology of Bacteria, Vol 2, Ecophysiology and Biochemistry, 3rd Edn, eds Dworkin M. Falkow S. Rosenberg E. Schleifer K. H. Stackebrandt E., 309–335.

[B67] Schink B. Montag D. Keller A. Müller N. (2017). Hydrogen or formate: alternative key players in methanogenic degradation. Environ. Microbiol. Rep. 9, 189–202. 10.1111/1758-2229.12524 28205388

[B68] Sedano-Núñez V. T. Boeren S Stams A. J. Plugge C. M. (2018). Comparative proteome analysis of propionate degradation by *Syntrophobacter fumaroxidans* in pure culture and in coculture with methanogens. Environ. Microbiol. 10.1111/1462-2920.14119 PMC5947623 29611893

[B69] Shimoyama T. Kato S. Ishii S. Watanabe K. (2009). Flagellum mediates symbiosis. Science 323, 1574–1574. 10.1126/science.1170086 19299611

[B70] Shrestha P. M. Rotary A. E. Summers Z. M. Shrestha M. Liu F. Lovley D. R. (2013). Transcriptomic and genetic analysis of direct interspecies electron transfer. Appl. Environ. Microbiol. 79, 2397–2404. 10.1128/AEM.03837-12 23377933 PMC3623256

[B71] Sieber J. R. Crable B. R. Sheik C. S. Hurst G. B. Rohlin L. Gunsalus R. P.. (2015). Proteomic analysis reveals metabolic and regulatory systems involved in the syntrophic and axenic lifestyle of *Syntrophomonas wolfei*. Front. Microbiol. 6:115. 10.3389/fmicb.2015.00115 25717324 PMC4324140

[B72] Sieber J. R. Mcinerney M. J. Gunsalus R. P. (2012). Genomic insights into syntrophy: the paradigm for anaerobic metabolic cooperation. Annu. Rev. Microbiol. 66, 429–452. 10.1146/annurev-micro-090110-102844 22803797

[B73] Sieber J. R. Sims D. R. Han C. Kim E. Lykidis A. Lapidus A. L.. (2010). The genome of *Syntrophomonas wolfei*: new insights into syntrophic metabolism and biohydrogen production. Environ. Microbiol. 12, 2289–2301. 10.1111/j.1462-2920.2010.02237.x 21966920

[B74] Tatusov R. L. Fedorova N. D. Jackson J. D. Jacobs A. R. Kiryutin B. Koonin E. V.. (2003). The COG database: an updated version includes eukaryotes. BMC Bioinformatics 4:41. 10.1186/1471-2105-4-41 12969510 PMC222959

[B75] Thauer R. K. (2012). The Wolfe cycle comes full circle. Proc. Natl. Acad. Sci. U.S.A. 109, 15084–15085. 10.1073/pnas.1213193109 22955879 PMC3458314

[B76] Trapnell C. Williams B. A. Pertea G. Mortazavi A. Kwan G. van Baren M. J.. (2010). Transcript assembly and quantification by RNA-Seq reveals unannotated transcripts and isoform switching during cell differentiation. Nat. Biotechnol. 28, 511–515. 10.1038/nbt.1621 20436464 PMC3146043

[B77] Ulrich L. E. Zhulin I. B. (2010). The MiST2 database: a comprehensive genomics resource on microbial signal transduction. Nucleic Acids Res. 38, D401–D407. 10.1093/nar/gkp940 19900966 PMC2808908

[B78] Wagner T. Koch J. Ermler U. Shima S. (2017). Methanogenic heterodisulfide reductase (HdrABC-MvhAGD) uses two noncubane [4Fe-4S] clusters for reduction. Science 357, 699–703. 10.1126/science.aan0425 28818947

[B79] Walker C. B. Redding-Johanson A. M. Baidoo E. E. Rajeev L. He Z. Hendrickson E. L.. (2012). Functional responses of methanogenic archaea to syntrophic growth. ISME J. 6, 2045–2055. 10.1038/ismej.2012.60 22739494 PMC3475374

[B80] Wang L. Wang S. Li W. (2012). RSeQC: quality control of RNA-Seq experiments. Bioinformatics 28, 2184–2185. 10.1093/bioinformatics/bts356 22743226

[B81] Wood G. E. Haydock A. K. Leigh J. A. (2003). Function and regulation of the formate dehydrogenase genes of the methanogenic archaeon *Methanococcus maripaludis*. J. Bacteriol. 185, 2548–2554. 10.1128/JB.185.8.2548-2554.2003 12670979 PMC152622

[B82] Worm P. Fermoso F. G. Stams A. J. Lens P. N. Plugge C. M. (2011a). Transcription of *fdh* and *hyd* in *Syntrophobacter* spp. and *Methanospirillum* spp. as a diagnostic tool for monitoring anaerobic sludge deprived of molybdenum, tungsten and selenium. Environ. Microbiol. 13, 1228–1235. 10.1111/j.1462-2920.2011.02423.x 21332622

[B83] Worm P. Stams A. J. Cheng X. Plugge C. M. (2011b). Growth-and substrate-dependent transcription of formate dehydrogenase and hydrogenase coding genes in *Syntrophobacter fumaroxidans* and *Methanospirillum hungatei*. Microbiology 157, 280–289. 10.1099/mic.0.043927-0 20884694

