# Supplementary material for: Concerted Metabolic Shifts Give New Insights Into the Syntrophic Mechanism Between Propionate-Fermenting Pelotomaculum thermopropionicum and Hydrogenotrophic Methanocella conradii
Source: Front Microbiol. 2018 Jul 9;9:1551. doi: 10.3389/fmicb.2018.01551 (PMC6046458; doi:10.3389/fmicb.2018.01551)
Supplement: Supplementary file 1 [file Table_1.PDF]

Supplementary Methods and Tables

Concerted metabolic shifts give new insights into the syntrophic mechanism between propionate-fermenting *Pelotomaculum thermopropionicum* and hydrogenotrophic *Methanocella conradii*

Pengfei Liu and Yahai Lu

This file contains:

1, Supplementary Methods

2, Supplementary Tables

1, Supplementary Methods

Amplification of intergenic regions of gene cluster for Fwd-Hdr-Mvh complex in M. conradii

DNA/RNA co-extraction, DNA digestion, cDNA synthesis and primer design were carried out as described in the main text. For the amplification of intergenic regions, primer pairs listed in Table S5 were used. Each 50-µl reaction mixture contained GoTaq® Flexi buffer 10 µl, $MgCl_2$, 2.5 mM, dNTPs, 10mM each 1µl, forward primer 0.5 µM, reverse primer 0.5 µM. Two microliter of DNA free RNA (r), cDNA (c) or genomic DNA (g) were used as templates. Thermo-cycling conditions were 95 °C for 2 min, 25 cycles of 95 °C for 30 s, 58 °C for 30 s, 72 °C for 1 min, 72 °C for 5 min.

2, Supplementary Tables

Table S1 Overview of RNA-seq reads and genome mapping.

Sample	Total reads (Million)	rRNA (%)	Reads mapped to genome (%)		Reads (Million) uniquely mapped to CDS[a]	
			M. conradii[b]	*P. thermopropionicum*[c]	*M. conradii*[b]	*P. thermopropionicum*[c]
PM1	27.5	0.10	0.63	n.a.	n.a.	20.2 (74%)
PM2	25.9	0.10	1.42	n.a.	n.a.	19.7 (76%)
PM3	27.5	0.09	0.67	n.a.	n.a.	21.2 (77%)
PM4	26.0	0.11	1.24	n.a.	n.a.	19.6 (76%)
PMC1	27.7	6.06	n.a.	n.a.	20.0 (71%)	1.9 (6.9%)
PMC2	27.6	5.47	n.a.	n.a.	18.9 (69%)	2.1 (7.8%)
PMC3	26.1	5.65	n.a.	n.a.	16.4 (63%)	3.4 (13.1%)
PMC4	25.6	2.75	n.a.	n.a.	18.3 (71%)	2.4 (9.5%)
MM1	25.7	7.14	n.a.	0.12	20.0 (78%)	n.a.
MM2	27.0	7.77	n.a.	0.11	21.0 (78%)	n.a.
MM3	26.1	5.63	n.a.	0.11	20.5 (79%)	n.a.
MM4	27.3	6.41	n.a.	0.11	21.3 (78%)	n.a.

Abbreviation: rRNA, ribosomal RNA; n.a., not applicable; PM, *P. thermopropionicum* monoculture; PMC, *P. thermopropionicum* and *M. conradii* coculture; MM, *M. conradii* monoculture.

[a]reads remaining after the most similar sequences between the two genomes were filtered out by cross mapping, ratio to total reads are shown in parentheses;

[b]*M. conradii* genome as reference; c, *P. thermopropionicum* as reference.

Table S2 Library strand specificity analysis by RSeQC.

Sample	1++,1--, 2+-,2-+	1+-,1-+, 2++,2--	Sample	1++,1--, 2+-,2-+	1+-,1-+, 2++,2--
P. thermopropionicum genome as reference					
PMCp1	0.1531	**0.8469**	PMp1	0.1315	**0.8686**
PMCp2	0.1584	**0.8416**	PMp2	0.1126	**0.8874**
PMCp3	0.1359	**0.8641**	PMp3	0.1133	**0.8867**
PMCp4	0.1756	**0.8244**	PMp4	0.1166	**0.8834**
M. conradii genome as reference					
PMCm1	0.0284	**0.9716**	MMm1	0.0148	**0.9852**
PMCm2	0.0371	**0.9629**	MMm2	0.0124	**0.9876**
PMCm3	0.0323	**0.9677**	MMm3	0.0126	**0.9874**
PMCm4	0.0372	**0.9628**	MMm4	0.0118	**0.9882**

PMp, dataset of *P. thermopropionicum* monoculture; PMCp, dataset of syntrophic coculture mapped to *P. thermopropionicum* genome; MMm, dataset of *M. conradii* monoculture; and PMCm, dataset of syntrophic coculture mapped to *M. conradii* genome.

For pair-end RNA-seq, there are two different ways to strand reads (such as Illumina ScriptSeq protocol):

1. 1++, 1--, 2+-, 2-+

read1 mapped to '+' strand indicates parental gene on '+' strand
read1 mapped to '-' strand indicates parental gene on '-' strand
read2 mapped to '+' strand indicates parental gene on '-' strand
read2 mapped to '-' strand indicates parental gene on '+' strand

2. 1+-, 1-+, 2++, 2--

read1 mapped to '+' strand indicates parental gene on '-' strand
read1 mapped to '-' strand indicates parental gene on '+' strand
read2 mapped to '+' strand indicates parental gene on '+' strand
read2 mapped to '-' strand indicates parental gene on '-' strand

Here, the vast majority was explained by the second way "1+-, 1-+, 2++, 2--", suggesting a strand-specific dataset.

Table S3 Pearson correlation of mapped reads between biological replicates.

PMp	1	2	3	4	MMm	1	2	3	4
1	1.000	0.987	0.987	0.988	1	1.000	0.989	0.989	0.989
2		1.000	0.995	0.995	2		1.000	0.989	0.990
3			1.000	0.994	3			1.000	0.994
4				1.000	4				1.000
PMCp	1	2	3	4	PMCm	1	2	3	4
1	1.000	0.960	0.973	0.958	1	1.000	0.988	0.991	0.986
2		1.000	0.957	0.952	2		1.000	0.983	0.981
3			1.000	0.969	3			1.000	0.991
4				1.000	4				1.000

PMp, dataset of *P. thermopropionicum* monoculture; PMCp, dataset of syntrophic coculture mapped to *P. thermopropionicum* genome; MMm, dataset of *M. conradii* monoculture; and PMCm, dataset of syntrophic coculture mapped to *M. conradii* genome.

Table S4 qRT-PCR primers for RNA-seq data validation used in this study.

Gene	Locus_tag	Gene products	Primer Name	Primer Sequence (5'-3')
**qRT-PCR primers for *M. conradii***				
16S rRNA	Mtc_r6	--	H16S-F	TACCGTGAGGCGTCCTGTTAAG
			H16S-R	GCCAGCAGTCTCCACAGTGTA
rpoA1	Mtc_2146	DNA-directed RNA polymerase, subunit A'	HRpoA1F	TGCGAGAACTGCGAGGTCTG
			HRpoA1R	TGTCCACTATCTTGCCGCTGAA
echE	Mtc_0802	Ech hydrogenase subunit E	echE-F	GGATAGAGCCTCTCCGCTTGAA
			echE-R	TAGCACACACCCTCTCCGAAAG
fwdB	Mtc_2470	Formylmethanofuran dehydrogenase subunit B	fwdB-F	GGAGGATGGCACCACTGAGTT
			fwdB-R	TCGCACGACGTTGAAGACCA
mcrA	Mtc_0908	Methyl-coenzyme M reductase, alpha subunit	mcrA-F	ATCTGGCTCGGCTCCTACATG
			mcrA-R	GGCTTGGTCGGCATCTTGTAC
fdhA	Mtc_2125	Formate dehydrogenase, alpha subunit (F420)	fdhA-F	AATATGCCTGCTCGCCCAGAA
			fdhA-R	TCGGAGCCTCTCGTAGGTCAT
acs1	Mtc_1904	Acetyl-CoA synthetases/AMP-(fatty) acid ligase	Hacs1F	AGATGACGGTGGCGATAGGC
			Hacs1R	ACAGGGCTTACCATCGGCATT
acs2	Mtc_2228	Acetyl-CoA synthetase/AMP-(fatty) acid ligase	Hacs2F	TGGACAGCCTGAGGTACATCAC
			HRpoA1R	CCGTCTCCGTCATCCAGTAGTT
**qRT-PCR primers for *P. thermopropionicum***				
16S rRNA	PTH_r004	--	P16SF	CGTGGATAACCTGCCTGTCAGA
			P16SR	TCCGTTACACCGCCAACTAGC
rpoC	PTH_0313	DNA-directed RNA polymerase, beta' subunit/160 kD subunit	PRpoCF	CGCAAGGTAATCGGCATCTGG
			PRpoCR	CTGACGGATCTGCTGGATGTTG
nuoF	PTH_2648	NADH:ubiquinone oxidoreductase, NADH-binding 51 kD subunit	PnuoFF	AGCAGGTGCGTTCGGTAACT
			PnuoFR	CGATAATTGCCTGACGGTCCAT
sdhA	PTH_1017	Succinate dehydrogenase/fumarate reductase, flavoprotein subunit	PsdhA1F	GGCGTTGTTCCTCTTGGCAAT
			PsdhA1R	GGGCTCGTAGTCGGGCATAA

mmcE	PTH_1361	Methylmalonyl-CoA mutase, N-terminal domain/subunit	PmmcEF	CACAGCCGAAGAAACCAACCA
			PmmcER	GCAACGCCAACCTTACCGATT
sfcA	PTH_2899	Malic enzyme	PsfcAF	GTCGCAGTTGCTGGACATACC
			PsfcAR	CCCGCTGATTACCACCCTGA
RAD55	PTH_0748	RecA-superfamily ATPase	PradF	GGTACGATCCTGCTCAAGTCCA
			PradR	ATGCCTGAGTCACCGATCTCG
pckA	PTH_1008	Phosphoenolpyruvate carboxykinase	PPckAF	TGTCGCCGAACGCCATACTT
			PPckAR	CGCCTTGTCCAGGTCGATTCT

Table S5 Primers for the amplification of intergenic regions of gene cluster for Fwd-Hdr-Mvh complex in *M. conradii*.

Region	Primer Name	Primer Sequence (5'-3')
fwdF::fwdG	inter1F	GCGGTCGTGGTCAATGAGGA
	inter1R	CGCCACAATACGAGCACTTCTT
hrC2::fwdF	inter2F	GCTTTACATCCCGCCAGTTGAG
	inter2R	AGCACGTCGAGCAGTACCATAT
hdrB3::hdrC2	inter3F	GCCGCTGTGGAAGGTGGTAA
	inter3R	TTAAGCCTCTCGTTCACGTCGT
hdrA2::hdrB3	inter4F	TCCGTTCTGTCACCTCCAGTTC
	inter4R	CGAGCAAGCAGCCACCACTA
mvhD::hdrA2	inter5F	GCGTGAACGATGCCATGTGTAA
	inter5R	TGAGCCTTCTCGGGTCAATGC
fwdB::fwdD::mvhD	inter6F	TGACGTTCGTCTCGGCATCC
	inter6R	GGCAGACCACATCCGTTACCA
fwdA::fwdB	inter7F	GCATAATCGGCGTAGAGGAGGA
	inter7R	TGCGACGTGCGAGTGGATAT
fwdc::fwdA	inter8F	TCTGGACTAATGCGGTGGATGG
	inter8R	CTTGGCTCCGATGTACTTGACC
