# Supplementary material for: Concerted Metabolic Shifts Give New Insights Into the Syntrophic Mechanism Between Propionate-Fermenting Pelotomaculum thermopropionicum and Hydrogenotrophic Methanocella conradii
Source: Front Microbiol. 2018 Jul 9;9:1551. doi: 10.3389/fmicb.2018.01551 (PMC6046458; doi:10.3389/fmicb.2018.01551)
Supplement: Supplementary file 3 [file Image_1.PDF]

Supplementary Figures

Concerted metabolic shifts give new insights into the syntrophic mechanism between propionate-fermenting *Pelotomaculum thermopropionicum* and hydrogenotrophic *Methanocella conradii*

Pengfei Liu and Yahai Lu

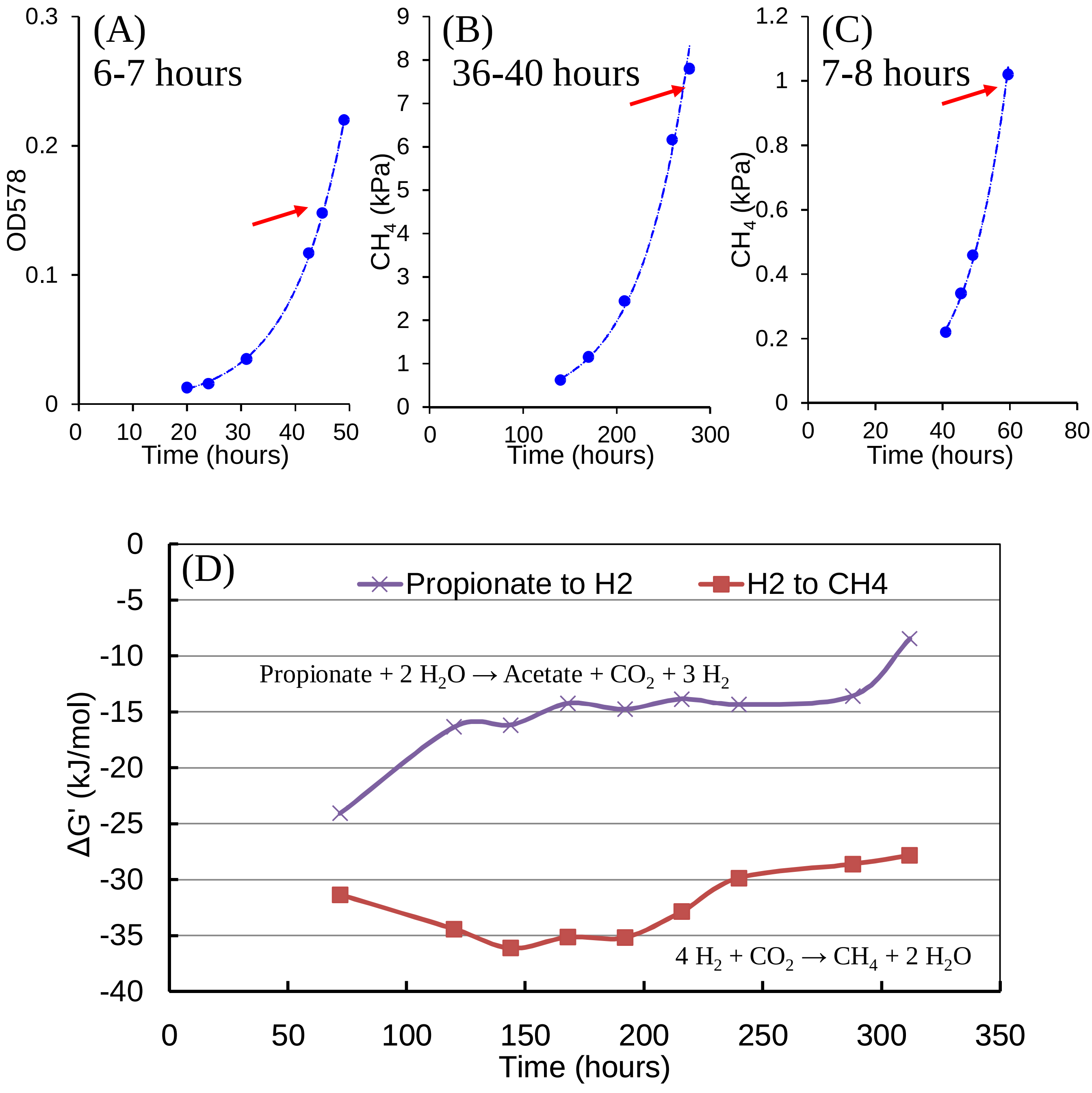

Figure S1 Growth curve of *P. thermopropionicum* monoculture on pyruvate (A), syntrophic coculture with *M. conradii* on propionate (B) and *M. conradii* monoculture on H_2/CO_2 (80/20, v/v, 170 kPa) (C) and the gibbs free energy change (*ΔG*) of propionate degradation and methane production in syntrophic coculture (D). Doubling times are shown for each culture and red arrows indicate sampling points for RNA-Seq analysis. For the calculation of gibbs free energy change, data from our previous work (Liu *et al*. 2014, Appl Environ Microbiol, 80: 4668–4676) were used.

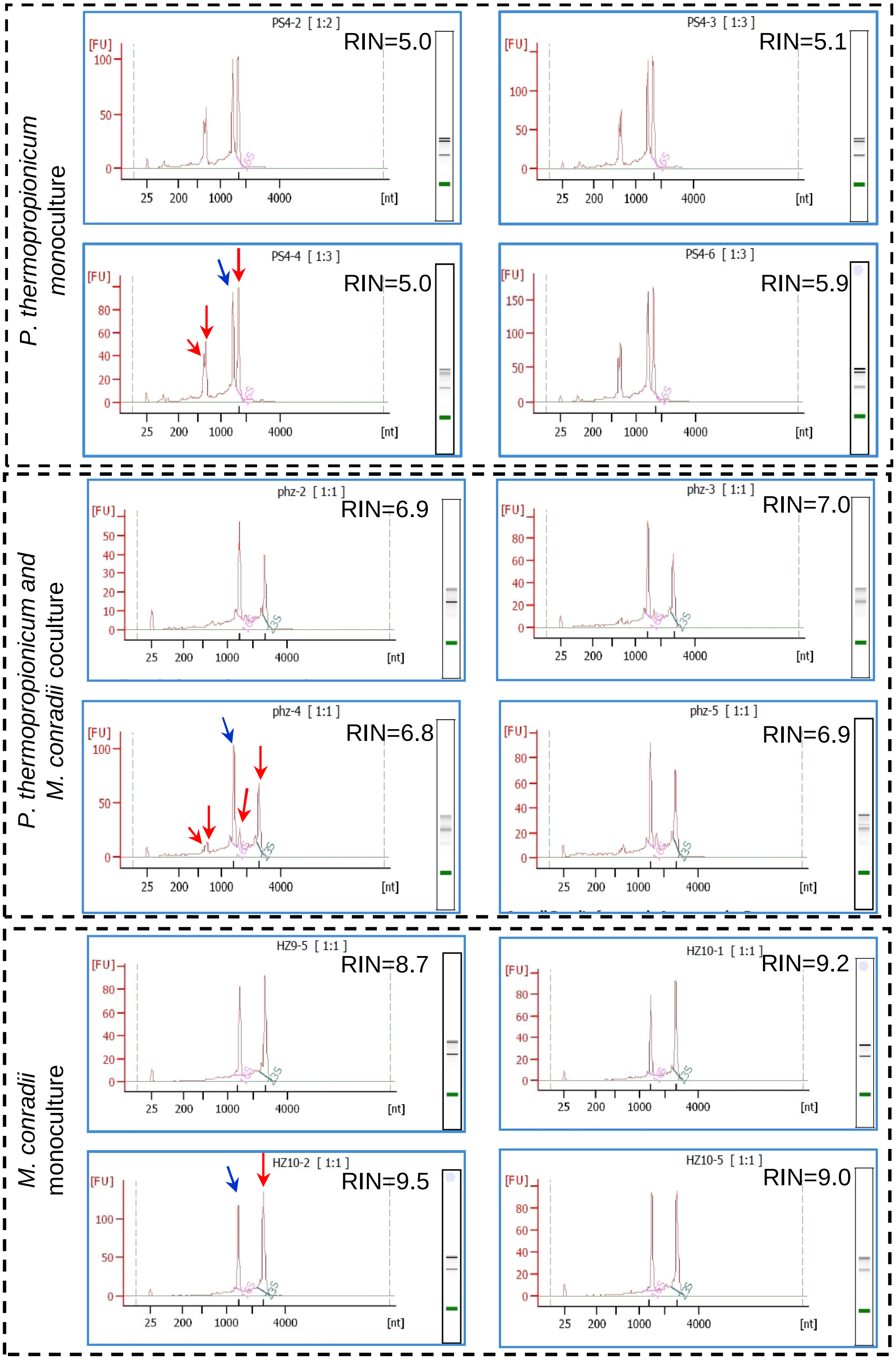

Figure S2 RNA integrity number (RIN) of total RNA from *P. thermopropionicum* monoculture (upper), coculture (middle) and *M. conradii* monoculture (lower). Each condition with four replicates. Red and blue arrows indicate 23S rRNA and 16S rRNA peaks, respectively. Due to the fragmentation of 23S rRNA of *P. thermopropionicum* (Kosaka et al., 2008, Genome Res 18: 442-448), 3 and 4 peaks of 23S rRNA were observed in *P. thermopropionicum* monoculture and syntrophic coculture, repectively. In coculture, the last peak of 23S rRNA was mainly derived from *M. conradii*.

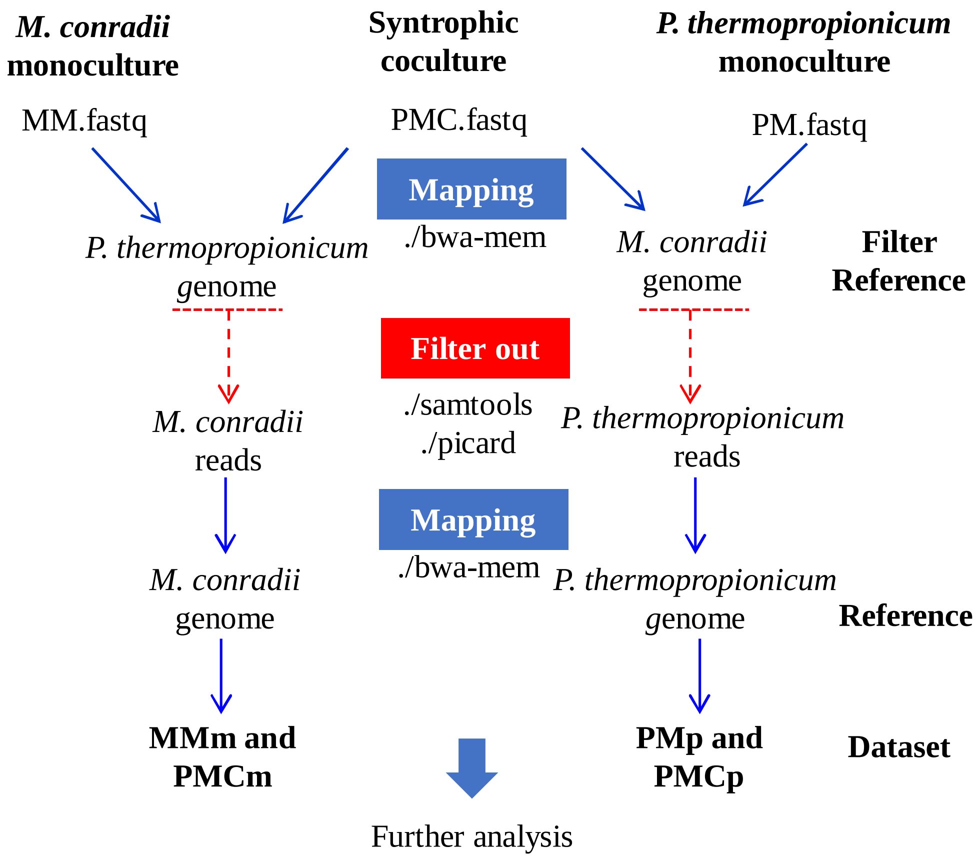

Figure S3 Cross mapping scheme for filtering out the most similar sequences between genomes of *P. thermopropionicum* and *M. conradii*. Abbreviations: PMp, dataset of *P. thermopropionicum* monoculture; PMCp, dataset of syntrophic coculture mapped to *P. thermopropionicum* genome; MMm, dataset of *M. conradii* monoculture; and PMCm, dataset of syntrophic coculture mapped to *M. conradii* genome.

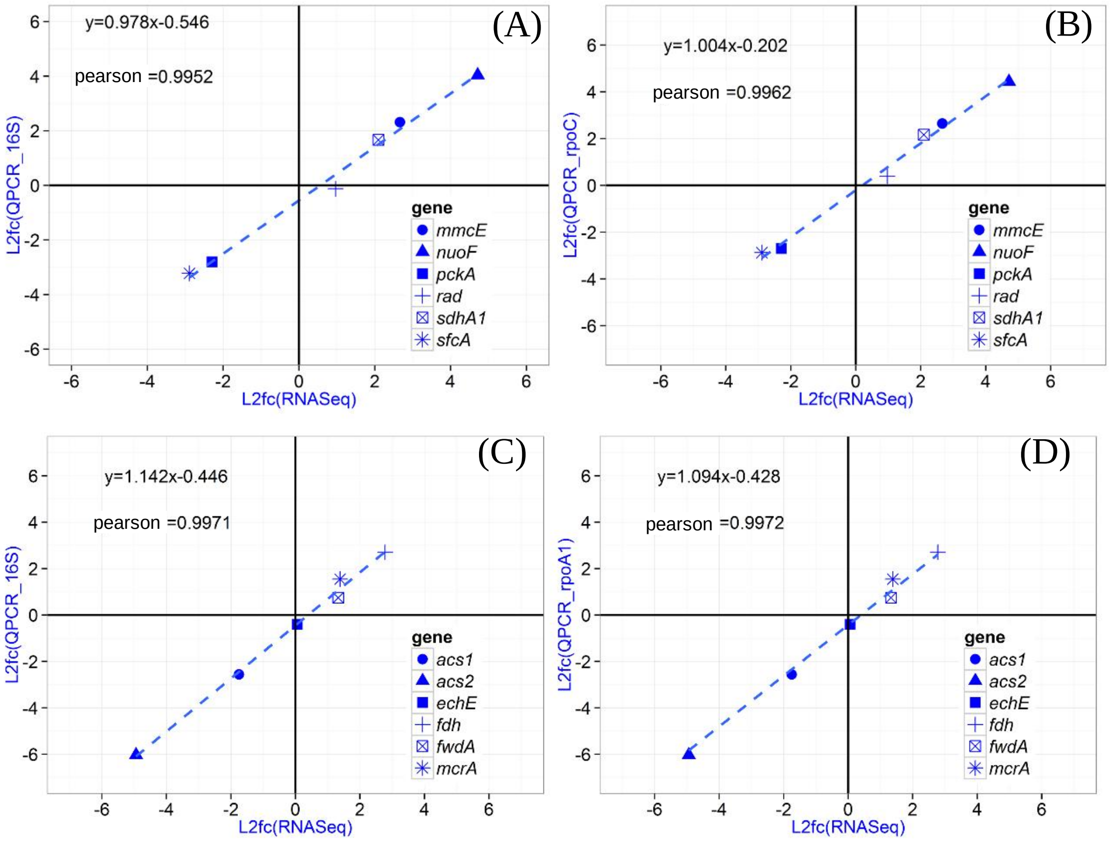

Figure S4 Verification of differentially expressed genes detected by RNA-Seq by qRT-PCR. Pearson correlation between RNA-Seq and qRT-PCR data for selected genes of *P. thermopropionicum*: (A), 16S rRNA as reference gene and (B), DNA-directed RNA polymerase beta subunit encoding gene (*rpoC*) as reference gene. Pearson correlation between RNA-Seq and qRT-PCR data for selected genes of *M. conradii*: (C), 16S rRNA as reference gene and (D), DNA-directed RNA polymerase A subunit encoding gene (*rpoA1*) as reference gene. L2fc, $\log_2$ fold change values.

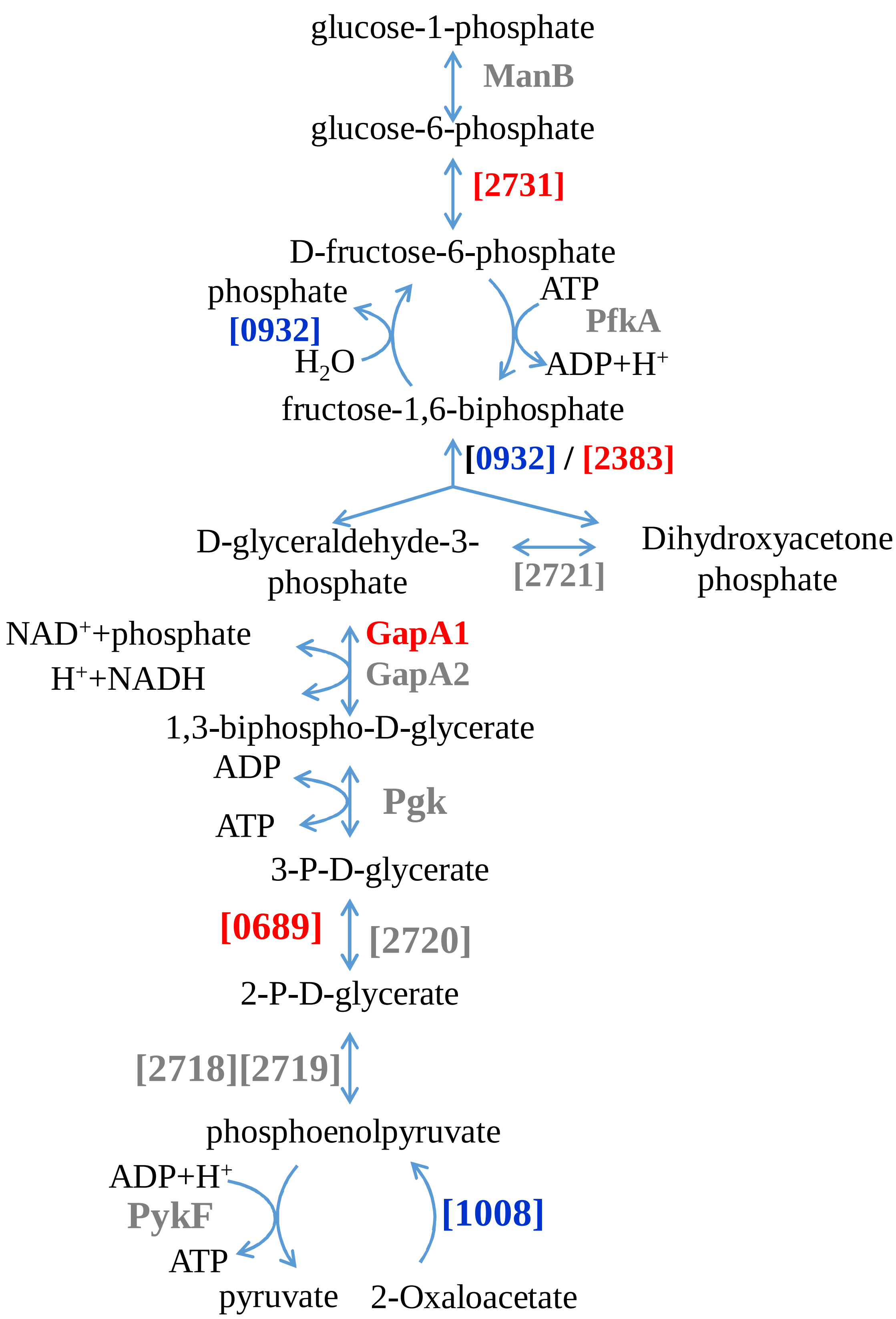

Figure S5 Differential expression of enzymes catalyzing each step of the glycolysis pathway in *P. thermopropionicum*. Relative changes (See Supplementary dataset Pt1 for details) in transcripts abundance during syntrophic growth are indicated by red (up) and blue (down) coloration. Grey coloration indicates statistically insignificant change (absolute $\log_2$ fold change < 1 or FDR ≥ 0.05).

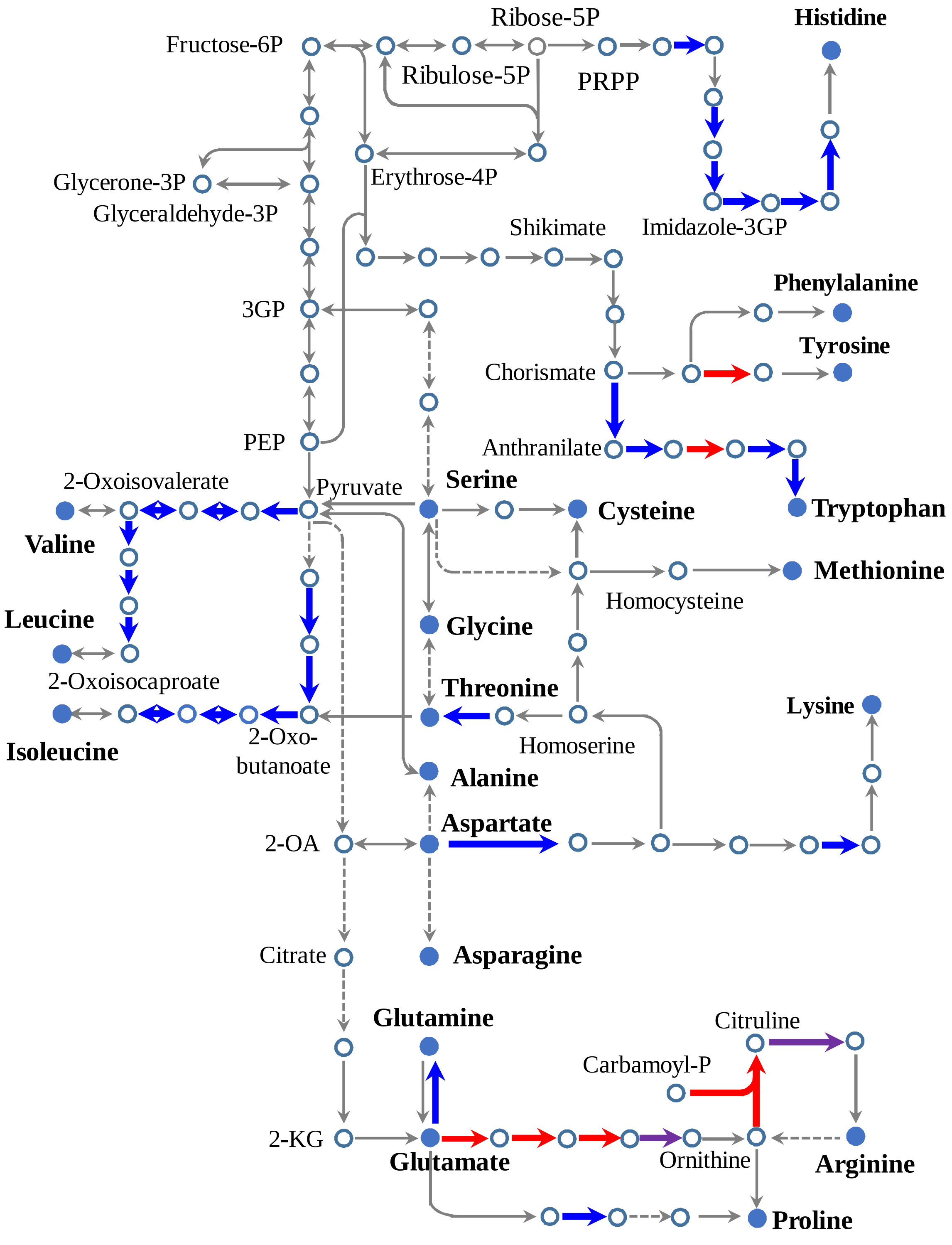

Figure S6 Differential expression of enzymes catalyzing each step of amino acids biosynthesis pathway in *P. thermopropionicum*. Relative changes (See Supplementary dataset Pt1 for details) in transcripts abundance during syntrophic growth are indicated by red arrows (up) and blue arrows (down). Enzyme names are not depicted. Amino acids are shown in bold. Grey arrows indicate statistically insignificant change (absolute $\log_2$ fold change < 1 or FDR $\geq$ 0.05). Dash arrows indicate steps missing in *P. thermopropionicum* genome annotation.

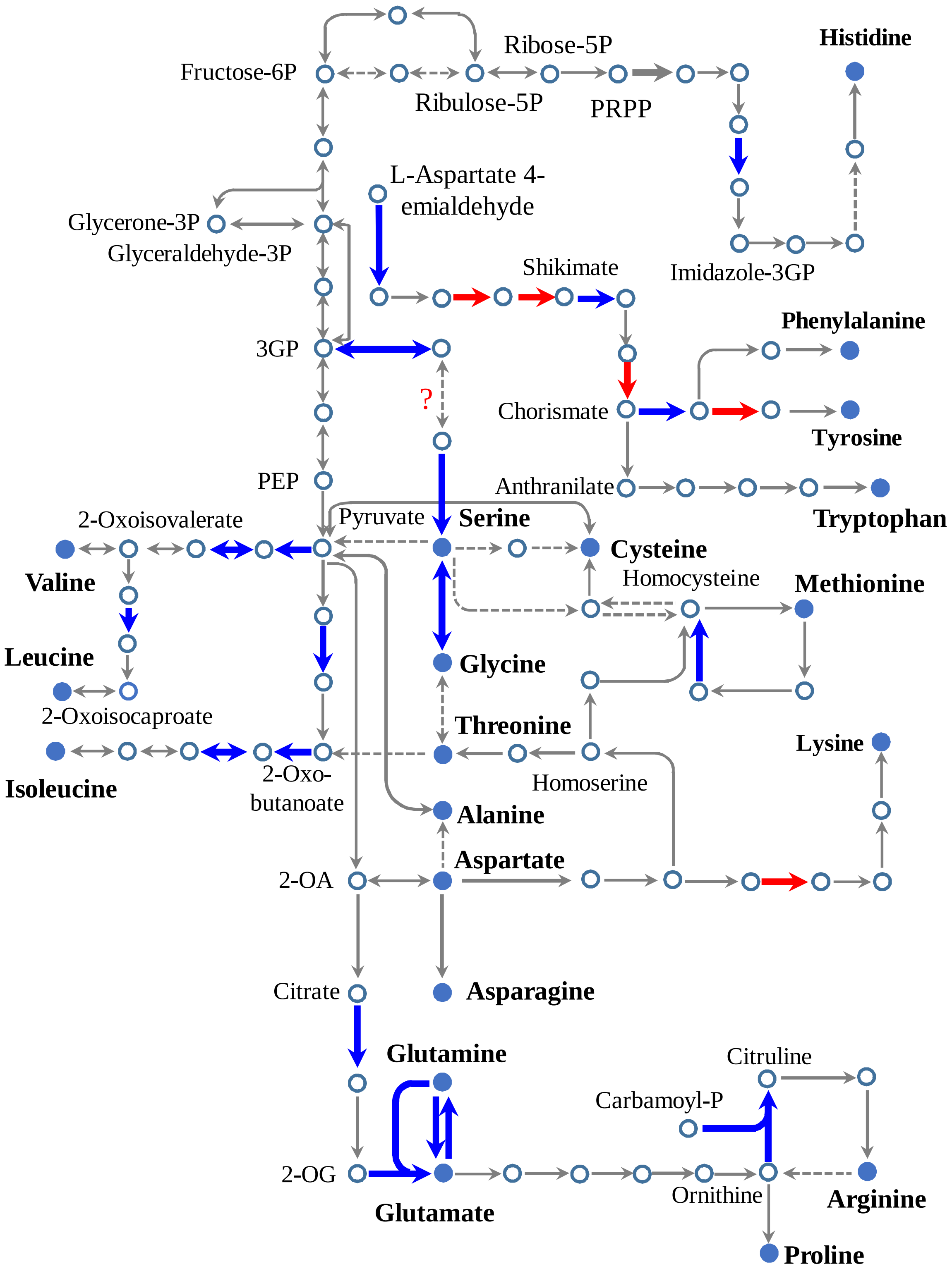

Figure S7 Differential expression of enzymes catalyzing each step of amino acids biosynthesis pathway in *M. conradii*. Relative changes (See Supplementary dataset Mc1 for details) in transcript abundance during syntrophic growth are indicated by red arrows (up) and blue arrows (down). Enzyme names are not depicted. Amino acids are shown in bold. Grey arrows indicate statistically insignificant change (absolute $\log_2$ fold change < 1 or FDR ≥ 0.05). Dash arrows indicate steps missing in *M. conradii* genome annotation.

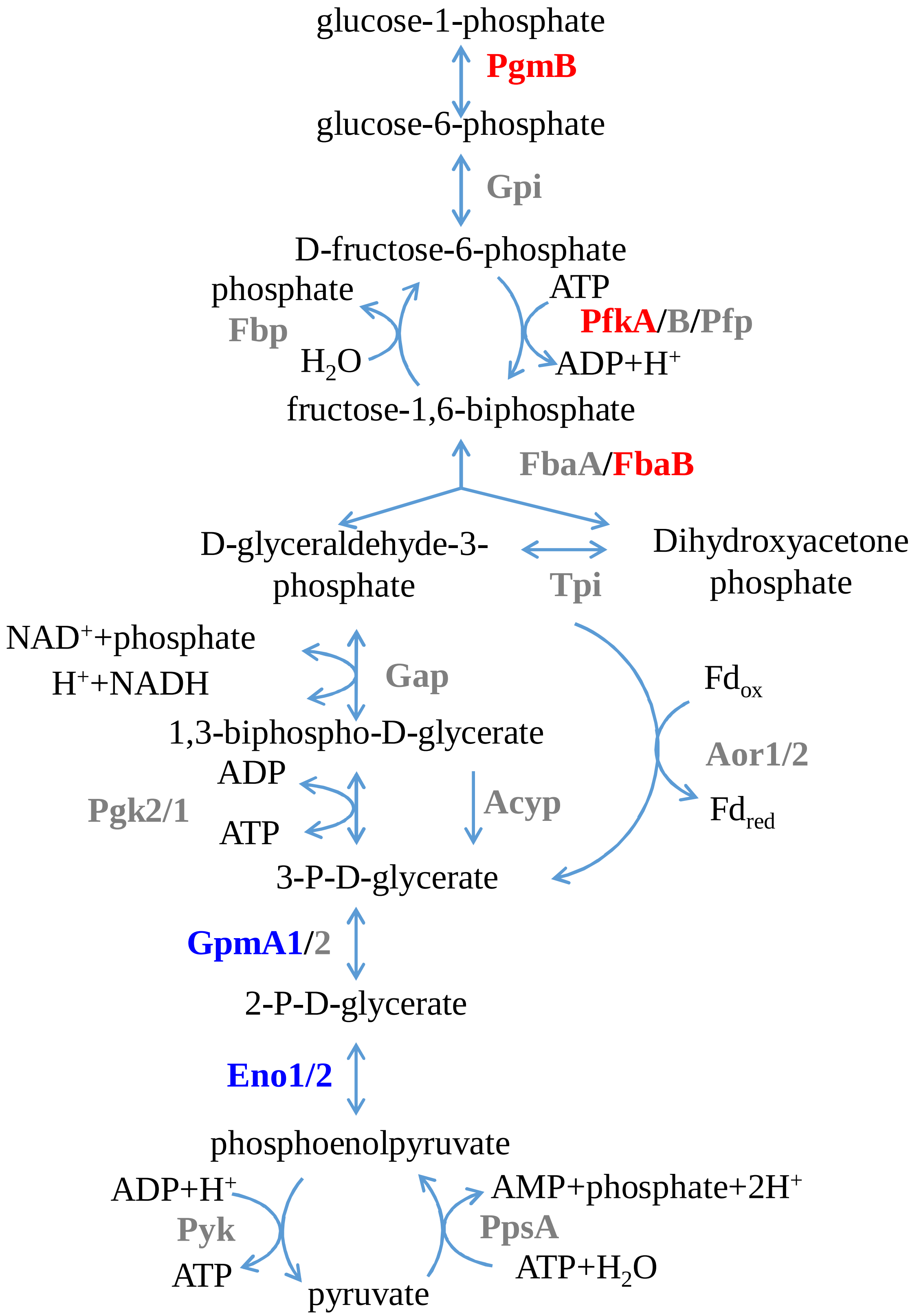

Figure S8 Differential expression of enzymes catalyzing each step of the glycolysis pathway in *M. conradii*. Relative changes (See Supplementary dataset Mc1 for details) in transcripts abundance during syntrophic growth are indicated by red (up) and blue (down) coloration. Grey coloration indicates statistically insignificant change (absolute $\log_2$ fold change < 1 or FDR ≥ 0.05).

(A)

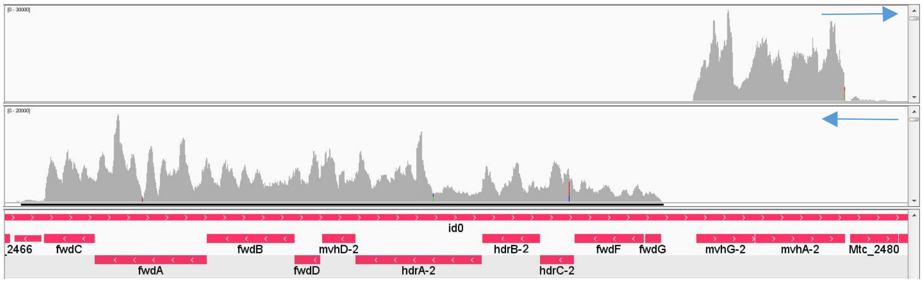

(B)

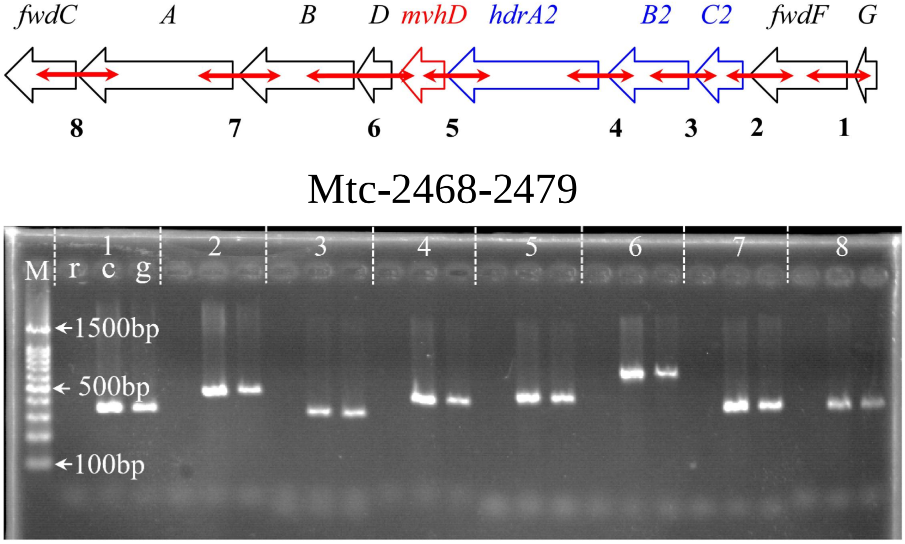

Figure S9 Visualization of the co-transcription of Fwd-Mvh-Hdr complex (A) and verification of the co-transcription of the Fwd-Mvh-Hdr coding genes by PCR (B). Upper panel of (B), gene cluster structure. Red arrows indicate the region the primers amplified. Lower panel of (B), gel electrophoresis of PCR products by using genomic DNA digested total RNA (r), cDNA (c) and genomic DNA (g) as template, respectively.
